# A subtype of cancer-associated fibroblasts with lower expression of alpha-smooth muscle actin suppresses stemness through BMP4 in oral carcinoma

**DOI:** 10.1038/s41389-018-0087-x

**Published:** 2018-10-05

**Authors:** Ankit Kumar Patel, Kavya Vipparthi, Venu Thatikonda, Indu Arun, Samsiddhi Bhattacharjee, Rajeev Sharan, Pattatheyil Arun, Sandeep Singh

**Affiliations:** 1 National Institute of Biomedical Genomics, Kalyani, India; 2 Tata Medical Center, Kolkata, India; 3 Present Address: German Cancer Research Center, Heidelberg, Germany

## Abstract

Cancer-associated fibroblasts (CAFs) demonstrate the characteristics of myofibroblast differentiation by often expressing the ultrastructure of alpha-smooth muscle actin (αSMA). However, heterogeneity among cancer-associated fibroblasts (CAFs), with respect to αSMA expression, has been demonstrated in several clinical studies of oral cancer. Like normal stem cells, stem-like cancer cells (SLCCs) are also regulated extrinsically by its microenvironment; therefore, we postulated that the heterogeneous oral-CAFs would differently regulate oral-SLCCs. Using transcriptomics, we clearly demonstrated that the gene expression differences between oral tumor-derived CAFs were indeed the molecular basis of heterogeneity. This also grouped these CAFs in two distinct clusters, which were named as C1 and C2. Interestingly, the oral-CAFs belonging to C1 or C2 clusters showed low or high αSMA-score, respectively. Our data with tumor tissues and in vitro co-culture experiments interestingly demonstrated a negative correlation between αSMA-score and cell proliferation, whereas, the frequency of oral-SLCCs was significantly positively correlated with αSMA-score. The oral-CAF-subtype with lower score for αSMA (C1-type CAFs) was more supportive for cell proliferation but suppressive for the self-renewal growth of oral-SLCCs. Further, we found the determining role of BMP4 in C1-type CAFs-mediated suppression of self-renewal of oral-SLCCs. Overall, we have discovered an unexplored interaction between CAFs with lower-αSMA expression and SLCCs in oral tumors and provided the first evidence about the involvement of CAF-expressed BMP4 in regulation of self-renewal of oral-SLCCs.

## Introduction

Oral cancer ranks among the top three within the South and Southeast Asian subcontinent and it is rapidly emerging across the globe^1,2^. In India, oral cancer accounts for over 30% of all cancer cases^3^. Gingivobuccal complex is among the most common sites for the incidence of oral cancer in India^4,5^. Despite the improvement in the standard treatment strategies, the 5-years survival rate has remained around 50% since decades^6,7^. This rate further drops for all patients who are detected with loco-regional metastasis^8,9^. Expression of several molecular markers on carcinoma cells has been of prognostic values; however, owing to the heterogeneity of carcinoma cells, their validity has been conflicting^10,11^.

Most cancers contain subpopulations of cancer cells with stem cell-like properties of unlimited self-renewal and differentiation to a state with limited proliferation ability. These stem-like cancer cells (SLCCs) are believed to drive malignancy by contributing towards therapeutic resistance, metastasis and relapse^12–15^. Gain in stem-like cancer cell population is found to have implications in tumor aggressiveness and poor clinical outcome^16–19^. Several in vitro and in vivo experiments have repeatedly demonstrated that the cells with higher activity of aldehyde dehydrogenase enzyme (ALDH) are enriched with stem-like cancer cells in oral cancer^20–22^. These cells were shown to express regulators of embryonic stem cell self-renewal factor, Oct4 and enhanced in vivo tumor forming ability^23–26^. Like normal stem cells, SLCCs may have the possibilities of retaining responsiveness and dependence to the external signals for their survival, growth and differentiation^14,27–30^. Therefore, ‘normal’ components of oral tumor microenvironment (TME), including fibroblasts, inflammatory cells and endothelial cells are gaining importance in their role in promoting tumor progression^31–33^. These cells reciprocate with SLCCs through direct cell to cell interaction or via several secretory factors^34,35^. Among several such factors; BMP4 is shown to act in specific spatiotemporal manner and demonstrates its role in embryonic developmental morphogenesis and shown to be essential for normal epidermal stratification^36,37^. The role of BMP4 is also emerging in oncogenesis, as reported in inducing differentiation of glioma and colorectal cancer stem-cells^35,38^.

Cancer-associated fibroblasts (CAFs) are the most abundant and critical cellular component of TME in a variety of solid tumors^33,39^. CAFs produce ultrastructure of alpha-smooth muscle actin (αSMA) and frequently demonstrate the characteristics of myofibroblast-differentiation^33,40^. Several studies have demonstrated a significant heterogeneity in the levels of αSMA-positive CAFs in oral tumors^41–43^. In these studies, it was demonstrated that oral cancer patients whose tumors had lower levels of stromal-αSMA expression had significantly longer disease free and overall survival^11,42,44,45^. Indeed, based on these studies, the concept of the existence of subtypes of CAFs is emerging in oral cancer. However, studies describing the precise molecular characterization of CAF-subtypes have been limiting. Here, we investigated CAF heterogeneity in primary cultures established from gingivobuccal oral tumors and postulated that the molecular differences among CAFs having variable levels of αSMA might cause distinct effects on the functional abilities of oral cancer cells.

We tested this hypothesis by immunohistochemical studies of primary tumor samples and molecular studies with primary cultures of several patient derived gingivobuccal oral CAFs. We found that at least two distinct subtypes of CAFs were present among all the established cultures as well as in primary tumors of gingivobuccal oral cancer. These subtypes of CAFs were distinct based on their gene expression and differently regulated the proliferation and stem-like behavior of oral carcinoma cells. CAFs with lower expression of αSMA correlated with lower frequency of oral-SLCCs and overall increased proliferation of cancer cells. Further, we provide evidences that these correlations are caused, in part, due to the differential expression of BMP4 by these CAFs.

## Results

### Isolation and characterization of CAFs from primary gingivobuccal-oral tumors

**Fig. 1:**
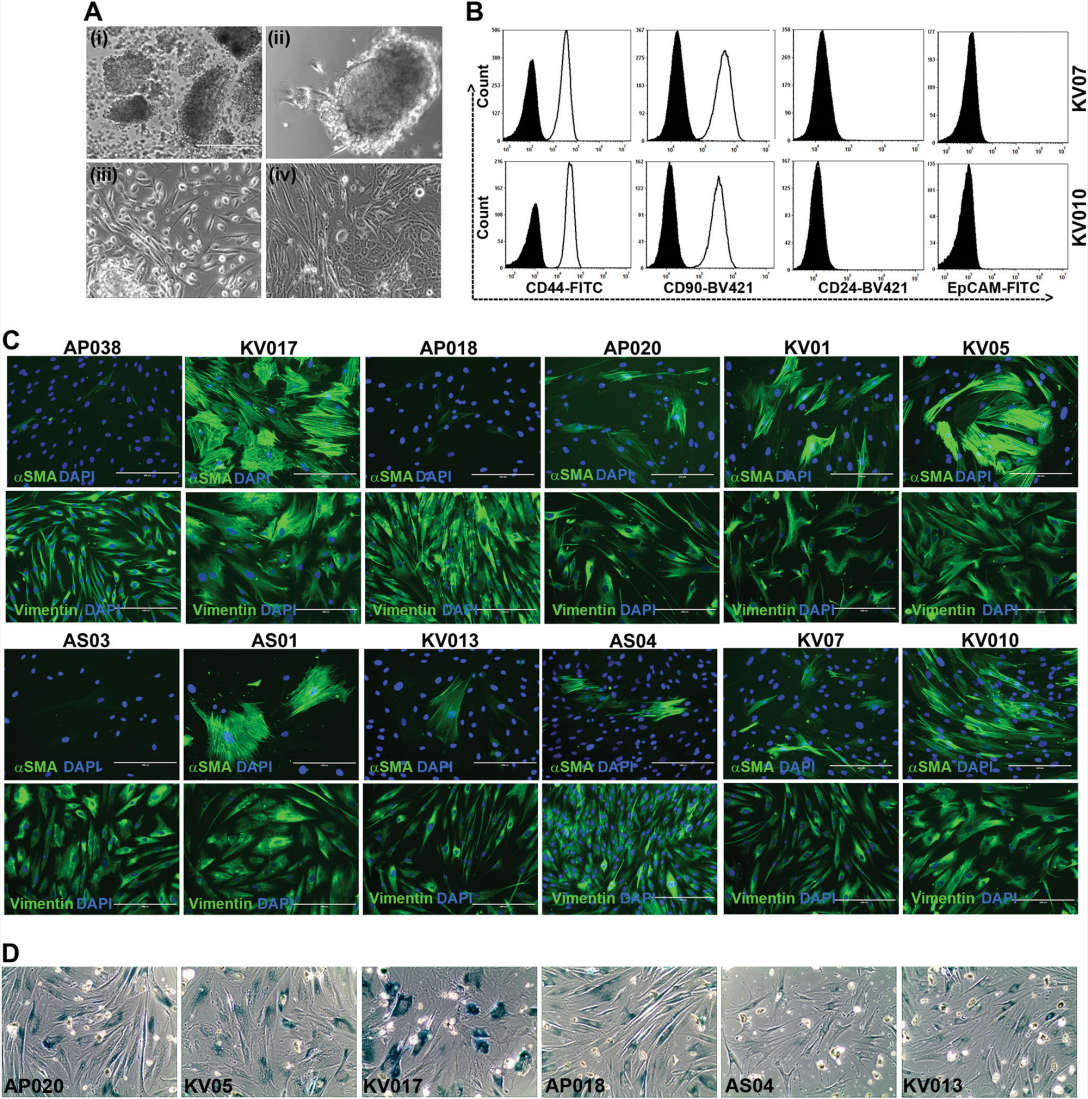
Establishment and characterization of oral-CAFs. **a** (i) OTOs plated over coated dishes. (ii and iii) CAFs and cancer cells growing from OTOs. (iv) CAFs and cancer cells were established from some samples as mixed culture. **b** Flow cytometry based phenotyping of established CAFs showing positive staining of CD44 and CD90, whereas negative for CD24 and EpCAM expression for two representative cultures. Filled histogram represents histogram for isotype control. **c** Immunofluorescence staining for αSMA (green) and vimentin (green) for the established CAFs cultures as indicated by its name. Staining demonstrated the heterogeneity in stress fiber of αSMA in established CAFs. DAPI is the nuclear counterstain. All Images are the representative of multiple fields taken at ×100 magnification. Bar represents 200 μm **d** Detection of senescence by SA-β-gal staining. Six different cultures of CAFs as indicated by its name, were plated on 8 well chamber slide and stained as mentioned in the text. Dark stained cells represented senescent cells. All Images are the representative of multiple fields taken at ×100 magnification

Oral tumor-derived CAFs were established as described in materials and methods. Oral tumor organoids (OTOs) were cultured over the collagen-coated surface. Within 1–3 weeks of incubation, CAFs started to grow from OTOs (Fig. 1a). All different established cultures were labeled by unique IDs, as indicated and characterized within 4–6 passages for expression of mesenchymal markers, CD44 and CD90. As shown in Fig. 1b, almost 99% of the cells showed homogeneous expression of CD44 as well as CD90. To test the chance of potential contamination of epithelial cells, the expression of EpCAM (CD326) as well as CD24 was assessed. All the CAF cultures were found to be negative for epithelial cells (Fig. 1b, Supplementary Figure S1). Interestingly, immunofluorescence staining of CAFs demonstrated heterogeneous expression of αSMA, where only certain cells were forming αSMA-positive stress fibers; and this number varied between different cultures of CAFs. Whereas, Vimentin was uniformly expressed between all the cells in each of these established cultures (Fig. 1c, Supplementary Figure S2). Heterogeneity among CAFs has also been linked to cellular senescence^46^. Therefore, we tested six different CAFs for SA-β-gal activity. In all the CAFs tested, cells demonstrated positive SA-β-gal activity with variation. KV017 showed higher SA-β-gal activity compared to KV05, AP020 and AP018, whereas AS04 and KV013 showed minimum activity (Fig. 1d).

### Matrix contraction ability of CAFs varies among different cultures

**Fig. 2:**
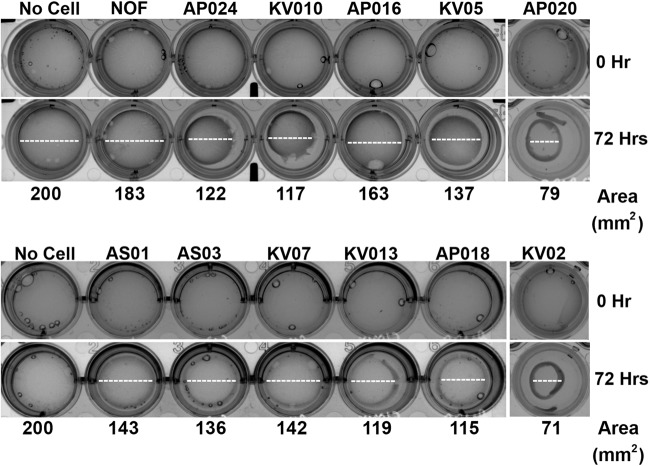
Collagen contraction assay of CAFs or normal-oral fibroblasts (NOF) were performed by mixing cells with collagen-I and matrigel matrix in 24-well dishes. Images were taken using Chemidoc imaging system before the plugs were allowed to contract and after 72 h of contraction. Dotted lines represent the diameter of the gel. Variation in contraction ability was observed among different CAFs

Since, expression of αSMA was found to be variable between different cultures of CAFs, we tested if these cells are variably activated. Collagen gel contraction ability was used as an indicator for activated nature of CAFs with matrix remodeling capability^47,48^. With discrete variability; all tested CAFs resulted in contraction of collagen gel, whereas normal gingival fibroblast (established from tissue obtained upon tooth extraction; Lonza) was not able to do so. Interestingly, irrespective of αSMA expression in these cells, we observed that the gel contraction ability was maximum for AP024, KV010, AP020 and KV02 CAFs, whereas AS01 and KV07 showed minimum ability, under similar conditions (Fig. 2).

### Gene expression differences segregated CAFs into two clusters

**Fig. 3:**
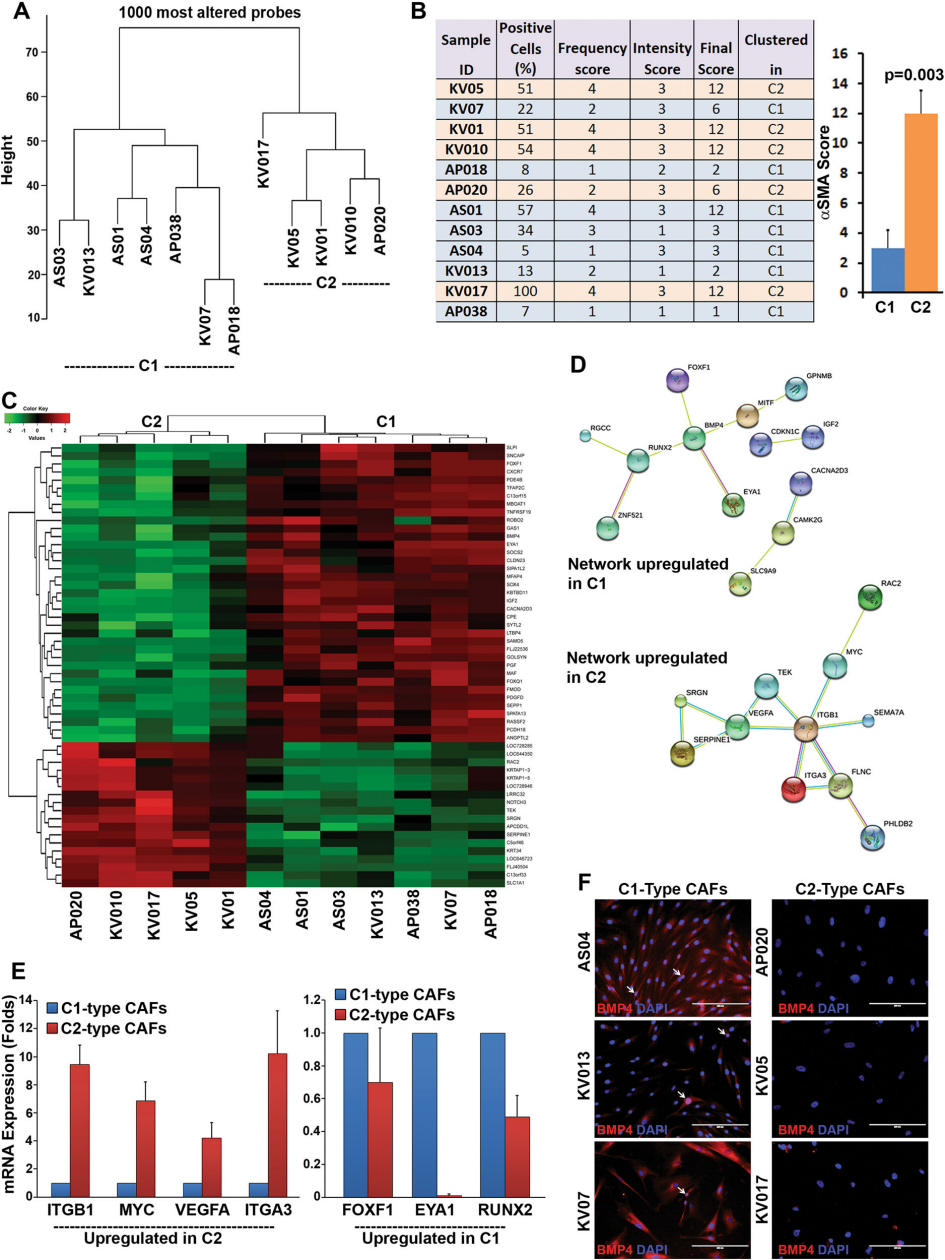
Gene expression analysis of CAFs by microarray. **a** Dendrogram showing unsupervised clustering of 1000 most variable probes. **b** Frequency of αSMA-positive cells and their level of expression were used to generate final αSMA-score for each of 12 CAFs used in the study. Bar graph represents the average αSMA-score for C1- and C2-type CAFs. Error bars show the standard deviation and *P* value was calculated by unpaired two-tailed *t* test. **c** Heatmap generated from the transcriptome analysis of RNA samples isolated from 12 different oral-CAFs. A total of 37 genes and 18 genes were up- and downregulated, respectively, in C1-type CAFs vs. C2-type CAFs (log fold change > 2; adjusted *P* < 0.05). **d** Analysis of protein-protein interaction network for genes upregulated (fold change > 2; adjusted *P* < 0.05) in C1- and C2-type CAFs using STRING-v10 software. Only connecting nodes are displayed. Pink edges represented experimentally determined known interactions. Blue and Green edges represent curated and text mining based interactions, respectively. **e** RNA samples from three different C1-type or C2-type CAFs were used to determine the levels of indicated genes by RT-PCR analysis. Fold change was calculated based on the average dCt value for CAFs in both clusters. **f** Expression of BMP4 (red) was determined by immunofluorescence staining for three different C1-type and C2-type CAFs. Arrows indicate some cells with nuclear expression of BMP4. DAPI is the nuclear counterstain. All Images are the representative of multiple fields taken at ×100 magnification. Bar represents 200 μm

Although, all different cultures of CAFs were propagated and maintained under similar conditions, they showed variation with respect to the frequency of cells expressing αSMA-positive stress-fibers and their ability to remodel collagen gel. Therefore, to explore the molecular basis of CAF-heterogeneity, we tested 12 different CAFs for transcriptomic differences, using gene expression microarray (Illumina). Very interestingly, unsupervised clustering using 1000 most variable probes resulted in segregation of all the CAFs into 2 major clusters (Fig. 3a). We named these clusters as C1 and C2 and the CAFs belonged to these clusters were labeled as C1-type CAFs or C2-type CAFs, respectively. We correlated these clustered-CAFs with their score of αSMA stress fiber expression (shown in Fig. 1c). The final score was obtained as given in Materials and Methods section. The data clearly demonstrated that the C2-type CAFs exhibited significantly higher score for αSMA as compared to C1-type CAFs (bar graph; Fig. 3b), without any significant difference between vimentin expression (Supplementary Figure S3). We next examined the differential gene expression between these CAFs from two clusters. Differentially expressed genes with adjusted *p* value of less than 0.05 and log fold change more than 2 were shortlisted and a heat map was generated (Fig. 3c). Complete list of differentially expressed genes (fold change > 2; adjusted *P* < 0.05) in C1-type CAFs verses C2-type CAFs is given in supplementary Table 1. Network analysis with STRING-v10 database, shortlisted significantly associated proteins among all upregulated genes from respective clusters (Fig. 3d). We validated the differential expression for some of the genes by real-time qPCR analysis. The level of expression correlated well between microarray and qPCR data. Three different CAFs of C1-type (AS04, KV013, KV07) and C2-type (AP020, KV05, KV017) were used to compare relative expression level of mRNA. Average dCt value from three C1-type CAFs and three C2-type CAFs was used to calculate ddCt and Fold changes (Fig. 3e). Further, differential expression of BMP4 was verified at protein level in C1-type (AS04, KV013, KV07) and C2-type (AP020, KV05, KV017) CAFs using immunofluorescence staining. Representative images are produced as Fig. 3f. Similar to the microarray results, BMP4 was highly expressed in all the tested C1-type CAFs. Interestingly, some of the cells were positive for nuclear expression of BMP4 (white arrows) in C1-type CAFs. Similar accumulation of BMP4 in the nucleus has been demonstrated earlier in a small proportion of colon cancer cells^49^.

Overall, these results confirmed that the transcriptome differences were indeed the basis of distinct clustering of the CAFs, which could also be correlated with myofibroblastic-differentiation score of these CAFs.

### C1-type CAFs modulated oral cancer cell proliferation and stemness

Distinctly clustered CAFs provided us the novel tool to understand the differences in reciprocal relationships between cancer cells and CAFs with variable degree of myofibroblast-differentiation. Thus, we further tested if the CAFs from two distinct clusters provide different effects on oral cancer cell properties.

**Fig. 4:**
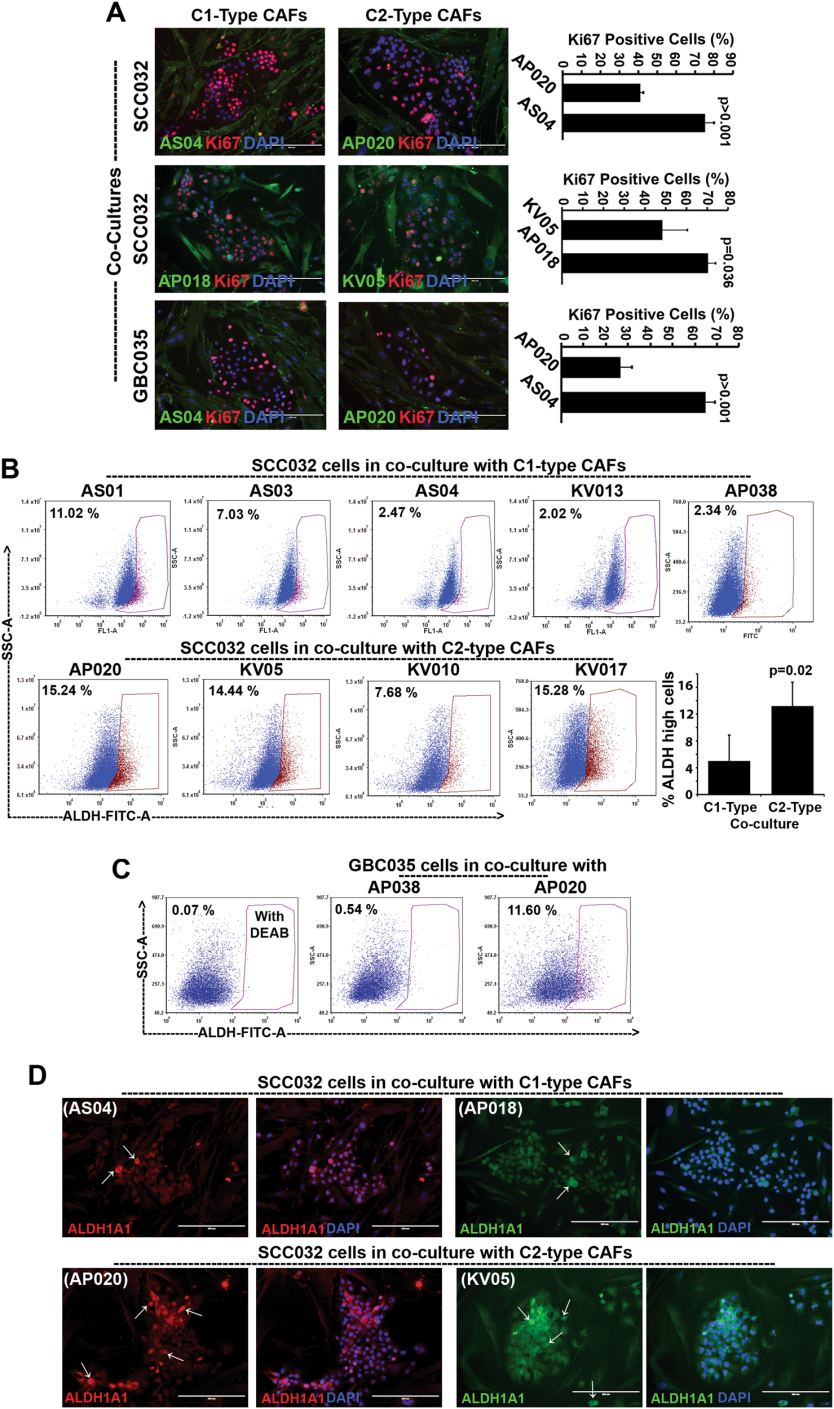
Proliferation and stemness properties of oral cancer cells upon co-culture with C1- or C2-type CAFs. **a** Immunofluorescence staining for Ki67 (red) as proliferation marker in CAF-cancer cell co-cultures. CD90 (green) was used to identify CAFs and nucleus was counterstained with DAPI (Blue). Ki67-positive nuclei of cancer cells and total number of cells were counted using ImageJ software and plotted for each co-cultures. Error bars show the standard deviation between (*n* = 3) biological repeats and *P* value was calculated by unpaired two-tailed *t* test. **b** Frequency of cells with high-ALDH activity was determined by Aldefluor assay for the CAF-cancer cell co-cultures using flow cytometry. Bar graph represents the average ALDH-high SCC032 cancer cells co-cultured with five different C1- and four different C2-type CAFs. Error bars show the standard deviation and *p* value was calculated by unpaired two-tailed *t* test. **c** Another oral cancer cell-GBC035 cells were co-cultured with indicated CAFs and results are presented as in. **d** Immunofluorescence staining for ALDH1A1 (red or green as indicated) as stemness marker in CAF-cancer cell co-cultures. Nucleus was counterstained with DAPI (Blue). Arrows indicate some cells with higher expression of ALDH1A1. All Images are the representative of multiple fields taken at ×100 magnification. Bar represents 200 μm

First, we checked its effect on cancer cells proliferation. SCC032 oral cancer cells were co-cultured with C1-type (AS04 or AP018) or C2-type (AP020 or KV05) CAFs for 5 days and checked for the number of epithelial cells with Ki67 expression (Fig. 4a). CAFs were distinguished by its positive staining for CD90 expression. Interestingly, SCC032 cells co-cultured with C1-type CAFs (AS04 or AP018) showed significantly higher percentage of Ki67 cells compared to its co-culture with C2-type CAFs (AP020 or KV05) (Fig. 4a). Similar result was observed when another oral cancer cell line (GBC035) was co-cultured with AS04 (C1-type CAFs) or AP020 (C2-type CAFs) (Fig. 4a).

We next tested the percentage of stem-like cancer cells with high aldehyde dehydrogenase activity (ALDH-Hi cells) using Aldefluor assay. SCC032 oral cancer cells were co-cultured with five different C1-type (AS01, AS03, AS04 or KV013, AP038) or four different C2-type (AP020, KV05, KV010, KV017) CAFs for 5 days and checked for the frequency of ALDH-Hi cells. CAFs were distinguished using CD90 expression and excluded from the analysis. Gating strategy for exclusion of CD90-positive population and negative control (DEAB treated sample) for Aldefluor Assay is shown as Supplementary Figure S4. Remarkably, compared to the co-culture with C2-type CAFs, SCC032 cells had significantly reduced frequency of ALDH-Hi cells upon co-culturing with C1-type CAFs (Fig. 4b). Similar result was observed when another oral cancer cell line (GBC035) was co-cultured with AP038 (C1-type CAFs) compared to AP020 (C2-type CAFs) (Fig. 4c).

To further confirm these observations, oral cancer cells (SCC032) were co-cultured with C1-type (AS04 and AP018) or C2-type (AP020 and KV05) CAFs for 5 days and immunofluorescence staining for ALDH1A1 was performed. Clearly, as compared to the co-cultures with C2-type CAFs; SCC032 cells co-cultured with C1-type CAFs had lower frequency of cells with high-expression of ALDH1A1 (shown as arrow, Fig. 4d).

### Observed effects of C1-type CAFs were in part mediated through BMP4

**Fig. 5:**
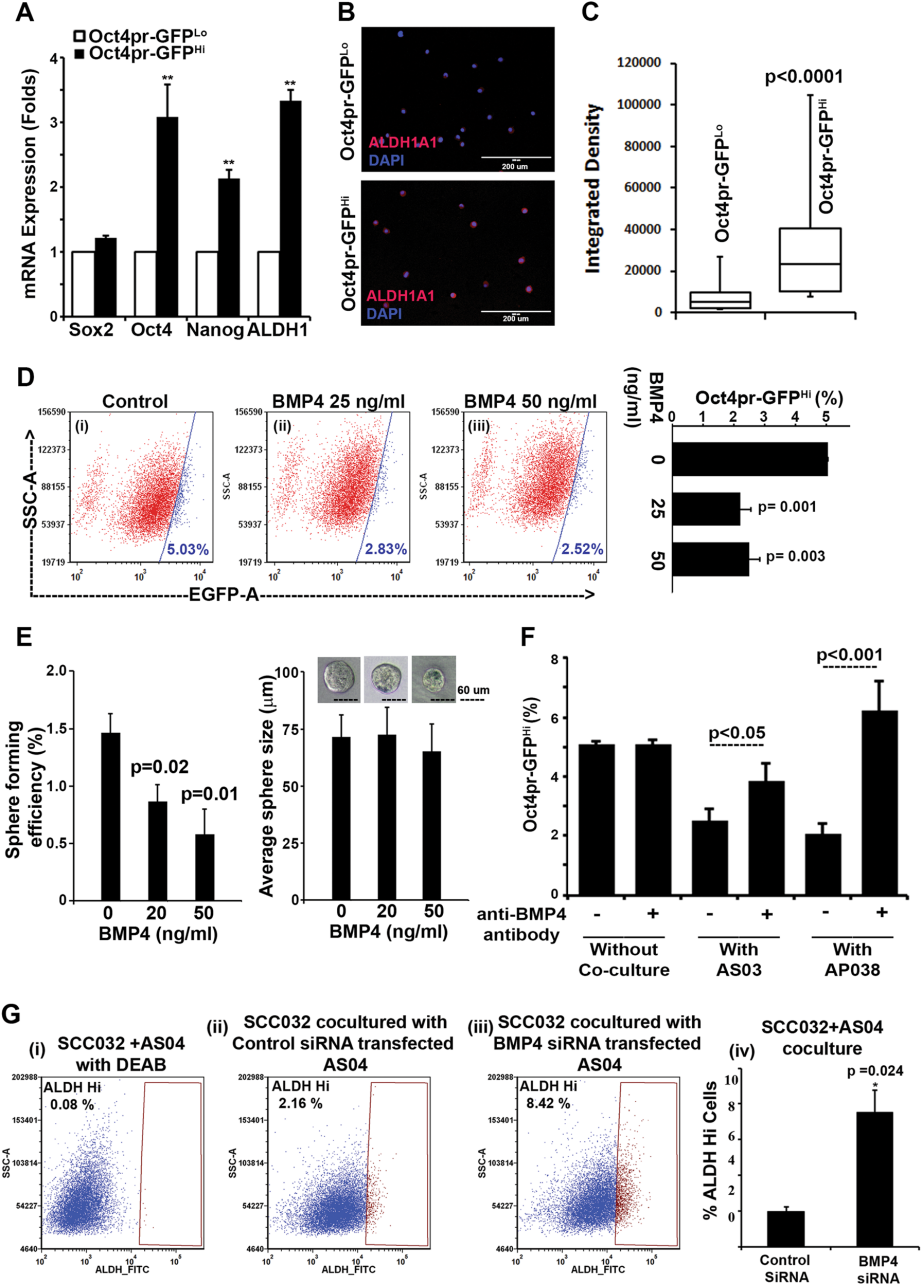
BMP4-mediated regulation of stemness. **a** RNA samples from sorted Oct4pr-GFPHi and Oct4pr-GFPLo cells were tested for mRNA expression of given genes. **b** ALDH1A1 (red) protein expression in these sorted cells were analyzed by immunofluorescence. **c** Box and whisker plot showing the integrated density of cells calculated using ImageJ software from 10 different fields. Error bars show the extreme quartiles of the intensities. **d** 29b-Oct4pr-GFP cells were grown in presence of 25 and 50 ng/ml BMP4 and the frequency of Oct4pr-GFPHi cells were determined by flow cytometry and shown as dot plots. Bar graph represents the average frequency of Oct4pr-GFPHi cells from three biological repeats. **e** SCC032 cells were tested for sphere formation in presence or absence of BMP4. All the spheres above 60 μm diameters were counted and plotted. Significant decrease in sphere formation efficiency was observed (i) without significant difference in average size of the spheres (ii). **f** Co-culture of C1-type CAFs (AS03 or AP038) with 029b-Oct4pr-GFP cells with or without BMP4 neutralizing antibody. Suppressive effect of C1-type CAFs was nullified by blocking BMP4 using BMP4 neutralizing antibody. **g** Co-culture of C1-type CAFs (AS04) with SCC032 cells after siRNA mediated knockdown of BMP4 and tested for ALDH Hi cells. DEAB was used as negative control for aldefluor assay (i). Suppressive effect of C1-type CAFs was nullified after BMP4-knowdown using BMP4 siRNA (iii) as compared to non-targeting siRNA (ii). The data are represented as bar graph (iv). For all the experiments, bar graph represents the average of minimum three biological repeats and error bars show the standard deviation. *P* value was calculated by unpaired two-tailed *t* test

To monitor the self-renewal growth, we generated a model using oral cancer cell line; SCC029b; as reporter cell line expressing EGFP under the control of Oct4-promoter (29b-Oct4pr-GFP cells). We collected top 5% eGFP-expressing cells (named Oct4pr-GFPHi) and compared with bottom 5% cells (Oct4pr-GFPLo). As shown in Fig. 5a, except Sox2, the expression of Oct4, Nanog, and ALDH1A1 was significantly higher (*p* < 0.01) in Oct4pr-GFPhi cells, supporting its stem cell-like property. We verified the expression of ALDH1A1 by immunofluorescence staining and representative images are shown in Fig. 5b. Expression of ALDH1A1 was quantified as integrated density value for intensity of ALDH1A1 immunofluorescence signal in each cell, using ImageJ software and plotted (Fig. 5c). On the basis of the median value; compared to Oct4pr-GFPLo cells, Oct4pr-GFPHi cells demonstrated, four fold higher ALDH1A1 levels (Fig. 5c). Collectively, enrichment of ALDH-Hi cells in Oct4pr-GFPHi population with higher expression of stem cell self-renewal factors implied that the 29b-Oct4pr-GFP cells would be suitable as alternate model to study the biology of stem-like oral cancer cells.

Since BMP4 was expressed by C1-type CAFs, we set to determine if the observed decline in frequency of oral stem-like cancer cells and increased proliferation of cells might be through induction of differentiation by BMP4 expressed by C1-type CAFs. Firstly, 29b-Oct4pr-GFP and SCC032 cells were treated with increasing concentration of BMP4 for 4 days and quantified for Ki67 expressing cells. However, we did not observe significant difference in Ki67-positive cells upon BMP4 treatment (data not shown). Interestingly, under similar conditions, BMP4 was able to suppress the frequency of Oct4pr-GFPHi cells, which is an indicative of loss of oral-SLCCs in response to BMP4 treatment (Fig. 5d). Further, to ascertain the role of BMP4 in suppression of self-renewal growth of oral-SLCCs, SCC032 cells were treated with 25 and 50 ng/ml of BMP4 under self-renewing conditions as sphere formation assay. As shown in Fig. 5e, significant decrease in the number of spheres was observed for BMP4 treated cells in dose dependent manner, without any effect on the size of the spheres.

To determine if the decrease in oral-SLCCs frequency in presence of C1-type CAFs was indeed mediated through BMP4, we performed co-culture experiment in absence or presence of neutralizing antibody against BMP4 (Cat.# MAB757; R&D Systems). 29b-Oct4pr-GFP cells were co-cultured with two different C1-type (AS03 and AP038) CAFs with or without anti-BMP4 antibody. Similar to the earlier observations, both the tested C1-type CAFs (AS03 and AP038) significantly reduced the frequency of Oct4pr-GFPHi cells (Fig. 5f). Interestingly, the frequency of Oct4pr-GFPHi cells was significantly higher, when the co-culture was performed in presence of anti-BMP4 neutralizing antibody. Importantly, the frequency of Oct4pr-GFPHi cells was not altered when anti-BMP4 antibody was added to the culture medium of 29b-Oct4pr-GFP cell alone.

To further substantiate our observations, we performed co-culture experiment after knockdown of BMP4 in C1-type CAFs (AS04). AS04 cells were transfected with non-targeting control siRNA or a specific siRNA against BMP4 (NM_001202; 5′-CAGCACUGGUCUUGAGUAU-3′ dTdT duplex). Forty-eight hours post-transfection, AS04-CAFs were co-cultured with SCC032 oral cancer cell for 4 days and tested for the frequency of ALDH Hi cells. Similar to the result obtained with BMP4 neutralizing antibody, knockdown of BMP4 in AS04 cells, significantly reduced the frequency of ALDH Hi cells compared to the negative control (Fig. 5g).

Overall, our data clearly demonstrated the determining role of BMP4 in exerting the suppressive effect of C1-type CAFs in oral cancer cells stemness, whereas proliferative effect was found to be independent of BMP4. Several growth factors upregulated in C1-type CAFs as provided in the differentially expressed gene list may be the regulators; and require additional experiments to understand the mechanism of increased proliferation.

### αSMA expression in stromal fibroblasts correlated with stemness and proliferation markers

**Fig. 6:**
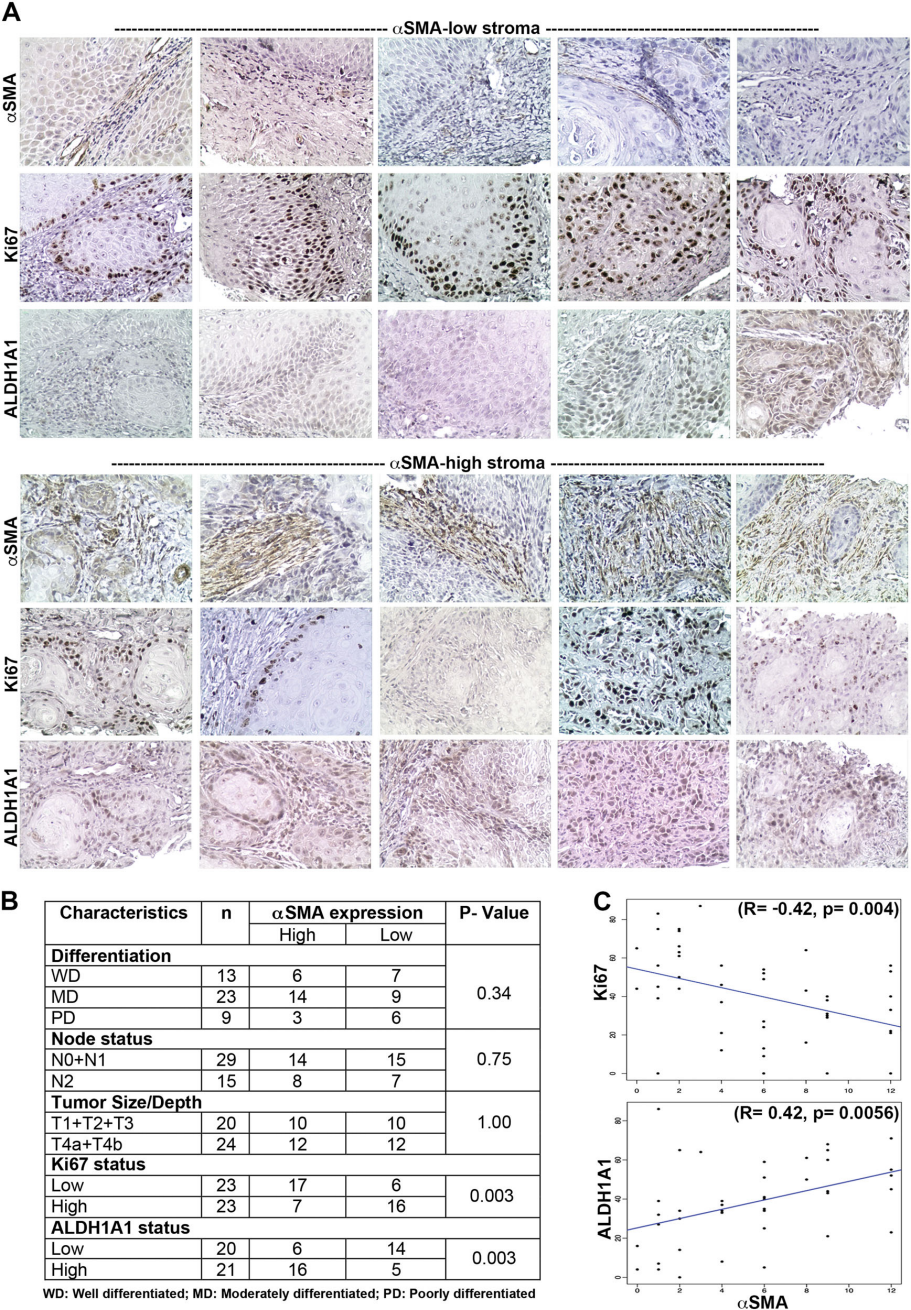
Immunohistochemical study of gingivobuccal oral tumor samples. **a** Oral tumor samples were stained for αSMA, Ki67 or ALDH1A1 and images were captured at 200x magnification. Staining for αSMA, Ki67 and ALDH1A1 for each tumor is shown for the representative samples from high or low αSMA group. **b** Table showing statistical correlation between αSMA staining and grade, node status, tumor size and Ki67 or ALDH1A1 staining for all the samples. **c** Regression analysis of αSMA with Ki67 or ALDHA1A expression. Proliferation index measured by Ki67 staining and frequency of oral-SLCCs measured by ALDH1A1 expression

History and the clinical characteristics of all the 46 gingivobuccal oral cancer patients included in the study are provided in Supplementary Table 2. These patient-samples were evaluated immunohistochemically for the presence of CAFs with αSMA expression. Representative images of the stained tissues are given in Fig. 6a. We observed heterogeneity in the levels of αSMA-positive CAFs in our samples. The staining index for αSMA was generated for the stromal fibroblasts. Among all the samples, staining index was high in 24 cases (~ 52%) and low in 22 cases (~48%). For all those samples where respective data were available, the correlation of αSMA-levels with histological grades, tumor size and lymph node metastasis status is summarized in Fig. 6b. Level of αSMA was not significantly different between these subgroups of gingivobuccal oral carcinomas (Fig. 6b).

Next, immunohistochemical staining for ALDH1A1 and Ki67 was performed. Representative images of the stained tissue sections are given in Fig. 6a. The staining index for ALDH1A1 and Ki67 was generated for all the cells present within the tumor islands. The median value for percentage of Ki67- or ALDH1A1-positive cells was determined. Samples which were having percentage positive cells with median value or above were considered high and rest were considered low. The staining index for Ki67 was high in 23 cases (50%) and low in 23 cases (50%). Interestingly, among all the low-Ki67 samples (*n* = 23), ~74% samples were having high score for αSMA, whereas among all the high-Ki67 samples (*n* = 23) only 30% samples were high for αSMA expression (Fig. 6b). Chi squared test suggested a significant association (*p* = 0.003) between αSMA and Ki67 among these samples. Similarly, among all the samples for which ALDH1A1 staining passed quality assessment (*n* = 41), staining index for ALDH1A1 was high in 21 cases (~51%) and low in 20 cases (~49%). Interestingly, among all the low-ALDH1A1 samples (*n* = 20), only 30% samples (6 of 20 samples) were having high score for αSMA, whereas among all high-ALDH1A1 samples (*n* = 21), ~76% samples (16 of 21 samples) were high for αSMA expression (Fig. 1b). Chi squared test suggested a significant association (*p* = 0.003) between αSMA and ALDH1A1 among samples.

Next, we performed regression analysis between αSMA and ALDH1A1 or αSMA and Ki67 and the results are presented in Fig. 6c. We found that the proliferation index as measured by percentage of Ki67-positive cells was significantly negatively correlated (*R* = −0.42, *p* = 0.004) with αSMA-score. On the basis of a linear model fit, number of Ki67-positive cells decreased (on an average) by 2.41% ([0.85%, 3.97%] is the 95% confidence interval) per unit increase in αSMA- score. This corresponded to increase of Ki67-positive cells by 16.87% ([5.95%, 27.79%] is the 95% confidence interval), as αSMA increases from 2 to 9. On the other hand, the frequency of oral-SLCCs as measured by ALDH1A1-expressing cells was significantly positively correlated (*R* = 0.42, *p* = 0.0056) with αSMA-score. On the basis of a linear model fit, ALDH1A1 score increased on an average by 2.37 ([0.79, 3.96] is the 95% confidence interval) per unit increase in αSMA-score. This corresponded to increase of ALDH1A1 by 21.33% ([7.11, 35.64] is the 95 % CI), as αSMA staining index increased from 2 to 9.

Overall, results with primary tumors demonstrated a significant concordance with our in vitro results where cancer cell proliferation and frequency of oral-SLCCs were significantly varying depending on the levels of myofibroblastic-αSMA-positive CAFs in gingivobuccal oral carcinoma.

## Discussion

Our results revealed that subtypes of CAFs exist in both primary cultures and human gingivobuccal oral tumors. αSMA expression in stromal-fibroblasts showed inverse relationship with proliferation of cancer cell population and a positive relation with frequency of stem-like cancer cell in gingivobuccal primary tumor samples and in vitro assays, in BMP4-mediated manner.

CAFs form a significant portion of the oral tumor stroma. Several previous studies have shown poor prognosis for patients, where stroma demonstrated myofibroblastic-αSMA-positive CAFs^11,42,44,45^. Therefore, differences in αSMA expression in stromal CAFs is very well documented, however, studies exploring the heterogeneity in CAFs and its probability of exhibiting diverse roles has been limiting in oral cancer. Our study revealed that all established CAFs were homogeneously positive for CD90 and CD44 expression; whereas, the expression of αSMA-positive stress fibers was variable among all the established CAFs. Similar to our observation, variable expression of αSMA has also been reported previously for the CAFs isolated from oral squamous cell carcinoma and linked with the genetic stability of the cancer cells in primary tumors. Genetically unstable OSCCs showed higher expression of αSMA in CAFs^50^. Variation in αSMA expression in CAFs have also been shown to be dependent on the senescence status of cells under in vitro conditions as well as in human oral tumors^46,51^. In agreement, we also observed higher β-gal activity in the cells with higher score for αSMA. This coincides with the notion that myofibroblast differentiation and senescence may co-occur during the program of fibroblast differentiation. However, this postulation needs further verification.

In a previous study, two subtypes of oral-CAFs have been reported based on the differential gene expression and secretome analysis between normal, dysplasia-associated or cancer-associated fibroblasts^52^. Surprisingly, the gene expression pattern of functionally most aggressive phenotype was found to be broadly similar to the normal fibroblasts. Authors have anticipated the systemic effect of tumor cells on adjacent normal tissue^52^. Here, our study reports the gene expression differences and similarities by comparing only the established CAFs without being dependent on gene-expression profile of normal fibroblast of adjacent tissue. We further acknowledge the fact that there are some disadvantages of studying cultured fibroblasts, including that there is absence of signals from other cell types present in tumor-microenvironment. However, here; all the CAF-cultures were established and propagated in the same conditions without any other cell-types, and thus had controlled condition for environmental signals. Therefore, our observed gene expression pattern is suggestive of the intrinsic and constitutive characteristics of the established CAFs.

Interestingly, based on the degree of similarity in gene expression, the established CAFs got grouped into two distinct clusters, named as C1-type and C2-type CAFs; which could also be correlated with the low or high αSMA-score of the CAFs, respectively. GO term enrichment analysis with DAVID functional annotation tool predicted ‘organ development’ (GO:0048513) and ‘cell migratory function’ (GO:0030335) as enriched biological process with the upregulated genes *MAF*, *BMP4*, *AEBP1*, *PGF*, *MITF*, *ARHGEF19*, *SOX4*, *LRIG3*, *ACACB*, *DCHS1*, *PCDH18*, *CDKN1C*, *EYA1*, *FOXQ1*, *CPE*, *PIR*, *FOXF1*, *RASSF2*, *TNFRSF19*, *ROBO2*, *TFAP2C*, *SEMA4D*, *PDGFD*, *GPNMB*, *RUNX2* in C1-type and *RAC2*, *SEMA7A*, *VEGFA*, *SERPINE1*, *TEK*, *ITGA3*, and *MYC* in C2-type CAFs, respectively. Very relevant to this finding, a recent report suggested that stromal cells similar to C2-type CAFs, with co-expression of SERPINE1 and αSMA at the invasive front of oral tumors could predict N-stage, presence of nodal metastasis, extracapsular spread and poor survival of the patients^42^. This is in accordance with our bioinformatics analysis, correlating the functional role of C2-type CAFs with invasion and metastasis of oral cancer.

Myofibroblastic phenotype of CAFs is shown to have better contractile function^48,53^. However, our results demonstrated that CAFs with high or low αSMA- score did not demonstrate any specific proportional ability for collagen-gel contraction in vitro. This is in accordance with a recent report suggesting that αSMA expression may not be the only determining factor to promote contraction of matrix^54^.

Demonstrating the distinct reciprocal effects, interestingly, our results provide evidence for the first time that in co-culture with C1-type of CAFs, oral cancer cells exhibited higher proliferation compared to the co-culture with C2-type CAFs. Additionally, the frequency of stem-like cancer cells was significantly lower in cancer cells upon co-culturing with C1-type CAFs. Therefore, these results clearly suggested that the Oral-CAF-subtype without myofibroblastic-differentiation (lower score for αSMA) were more supportive for cellular proliferation but less conducive for the self-renewal growth of stem-like cancer cells under in vitro conditions. Decreased frequency of oral-SLCCs in presence of C1-type CAFs was mediated through BMP4 expression. Activated BMP4 signaling is previously shown to differentiate stem-like cancer cell population in gliomas and colorectal cancer, suggesting it to be key regulator of human tumorigenesis^55,56^.

Importantly, our in vitro results were in complete concordance with in situ expression of αSMA, ALDH1A1 and Ki67 in gingivobuccal oral tumor samples. Even though, our sample size was small but a statistical test could be performed, which suggested a significant correlation between these markers. To the best of our knowledge, we are first to demonstrate the correlation between stromal expression of αSMA and the frequency of stem-like cancer cells in oral tumors. Taken evidences from our in vitro experiment, we can suggest that the low frequency of oral-SLCCs in tumors with lower αSMA (C1-type CAFs) may be a result of BMP4-mediated suppression of self-renewal. Also, lower frequency of oral-SLCCs may be one of the reasons for better survival of the patients having lower αSMA in tumor stroma. However these suppositions warrant further exploration.

**Fig. 7:**
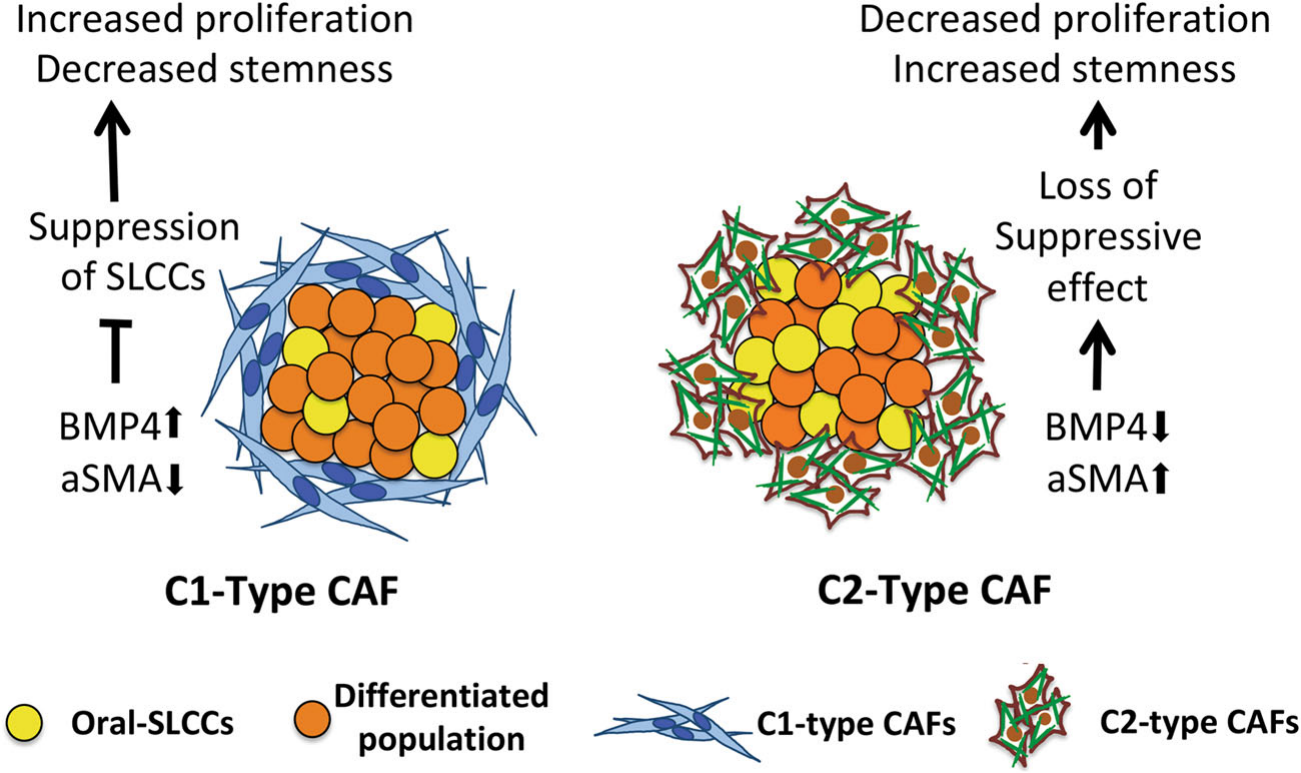
Model of tumor-stroma crosstalk. The functional heterogeneity in CAFs and its distinct reciprocal interaction with oral cancer cells is demonstrated. Oral-CAFs may be of two subtypes based on their gene expression and positivity for αSMA expression. CAFs with lower-αSMA score (C1-type CAFs), express higher levels of BMP4 and were associated with higher proliferation of oral cancer cells and lower frequency of oral-SLCCs. C1-type CAFs suppressed the self-renewal growth of oral-SLCCs in BMP4 dependent manner. On the other hand, CAFs with higher-αSMA score (C2-type CAFs), showed lower levels of BMP4 expression and were associated with lower proliferation of oral cancer cells with higher frequency of oral-SLCCs

In conclusion, we have characterized CAFs from gingivobuccal-oral cancer as two functionally distinct subtypes in both established cell cultures and primary human tumor samples. These subtypes were correlated with low- or high-αSMA-score and termed as C1-type or C2-type CAFs. Study is indicating that a small set of differentially expressed genes may be responsible for their distinct functions in oral tumors. Overall, it may be postulated that the C2-type CAFs are the result of tumor microenvironment evolution, which produces CAFs with myofibroblast phenotype (Fig. 7). Because, the resultant CAFs with downregulated BMP4 expression provide more conducive environment for deregulated self-renewal of oral-SLCCs, this evolution may be essential for tumor progression. Therefore, understanding the cellular mechanisms of BMP4-mediated regulation of self-renewal and differentiation of oral-SLCCs and its potential use as differentiating agent may lead to innovative interventions against gingivobuccal oral cancers.

## Materials and methods

### Generation of oral tumor organoids (OTOs) and establishment of cancer-associated fibroblasts cultures

Institutional ethics committee of National Institute of Biomedical Genomics and institutional review board of Tata Medical Center, India approved this study (EC/GOVT/01/12). Informed written consent was taken from patients before their inclusion in the study. Samples procured from 2014 to 2017 are included in this study. Samples were minced into small pieces under sterile conditions and dissociated using enzyme mix (Deoxyribonuclease 1 and Collagenase/Hyaluronidase; StemCell Technologies). Small tumor pieces were plated over poly-d-Lysine (Sigma Aldrich) and collagen-I (Stem cells Technologies) coated culture plates in DMEM/F12K (1:1) with 10% FBS (Thermo) and 1× Insulin Transferrin Selenium (ITS) (Thermo). Fibroblasts grown from the tissues were dissociated and cultured separately, over freshly coated cell culture plates.

With this protocol, primary culture of GBC035 oral cancer cells was established from tumor site of buccal mucosa. These cells were maintained in DMEM/F12K (1:1) with 10% FBS and 1× ITS. Oral cancer cell line SCC032 and SCC029b; established from retromolar trigone and buccal mucosa, respectively, were kindly provided by Dr. Susanne M. Gollin, University of Pittsburgh, USA. For all co-culture experiments, CAFs and oral cancer cell lines were plated at a ratio of 3:1 for 5 days in DMEM/F12K (1:1) supplemented with 1% FBS and 0.1× ITS. Cells were routinely tested for mycoplasma contamination using Myco Alert kit (Lonza; Cat No. LT07-418).

### Flow cytometry for cell surface markers and Aldefluor Assay

Flow cytometry was performed as described earlier^57^. Briefly, single-cell suspension was prepared using Accutase cell dissociation reagent (Thermo) and incubated with indicated antibody. After washing, cells were treated with Propidium Iodide (Sigma) to exclude dead cells. Isotype-matched IgG was tested for demonstrating antibody specificity. Aldefluor assay was performed according to manufacturer’s instructions (StemCell Technology) and as mentioned previously^58^. Flow cytometry data were acquired using BD Accuri C6 or BD FACS Aria Fusion cytometer and analysis was done with FCS Express 5 (DeNovo Software).

### Establishment of 29b-Oct4pr-GFP cells

We established a stable culture of SCC029b cells as a reporter cell line for monitoring Oct4 expression. SCC029b cells were transfected with phOct4-EGFP plasmid (Plasmid #38776; Addgene) using Lipofectamine 2000 reagent (Thermo) following manufacturer’s instructions. Cells were selected using 500 μg/ml G418 (Thermo) for 3 weeks. From all G418 resistant cells, bright EGFP-positive cells were sorted by FACS and allowed to regrow and analyzed for EGFP expression. The resultant culture showed the process of differentiation, resulting in a mixed culture of both bright- and dim-EGFP-positive cells. This culture was named as 29b-Oct4-eGFP and maintained with 50 μg/ml G418.

### Statistical analysis

*Χ*^2^ test and regression analysis was performed for testing statistical significance for the scores obtained for immunohistochemical staining of αSMA, ALDH1A1 and Ki67 from primary tumors. We checked for normality of the ALDH1A1 score and ki67 percent values using a normal Quantile-Quantile (QQ) plot. The deviations from normality were not significant (Shapiro Wilk’s test for normality, *p* = 0.54 and *p* = 0.5 for ALDH1A1 and Ki67 percent, respectively). Unpaired *t* test was performed for comparing two groups for all in vitro experiments. Data were considered statistically significant when the *p* value was less than 0.05.

## Electronic supplementary material

Supplementary figure legends

Supplementary Methods

Supplementary Table 1

Supplementary Table 2

Supplementary Figure S1

Supplementary Figure S2

Supplementary Figure S3

Supplementary Figure S4
