# Supplementary material for: A subtype of cancer-associated fibroblasts with lower expression of alpha-smooth muscle actin suppresses stemness through BMP4 in oral carcinoma
Source: Oncogenesis. 2018 Oct 5;7(10):78. doi: 10.1038/s41389-018-0087-x (PMC6172238; doi:10.1038/s41389-018-0087-x)
Supplement: Supplementary file 1 — Supplementary figure legends [file 41389_2018_87_MOESM1_ESM.docx]

**Supplementary Figure S1:** Expression of CD44, CD90, CD24 and EpCAM on patient-derived CAFs; analysed by flow cytometry. All the tested CAFs showed positive staining for CD90 and CD44.The dark area under the histogram showing the isotype IgG, and the blank area represents either of the markers (CD90, CD44, CD24 and EpCAM). All the tested CAFs showed negative staining for CD24 and EpCAM indicating that the established CAFs were pure cultures without contaminating epithelial cancer cells. (NA: Not assessed)

**Supplementary Figure S2:** Immunofluorescence images of CAFs. The labelled CAFs were immunostained for αSMA and vimentin. The upper panel shows the expression of αSMA for respective CAFs, αSMA: green. The lower panel shows the expression pattern of Vimentin for respective CAFs shown in green. Nucleus was counterstained with DAPI. Images were captured in 20x magnification in EVOS-FL microscope.

**Supplementary Figure S3:** Expression of Vimentin by C1-type or C2-type CAFs. Expression of vimentin was quantified by ImageJ software. Integrated density was calculated from 3 independent images of each CAFs and average integrated density was calculated for each subtype. Student *t* test was performed to for statistical significance.

**Supplementary Figure S4:** Aldefluor assay estimating the frequency of cells with higher activity of aldehyde dehydrogenase (ALDH) in CAFs-cancer cell co-culture conditions was analysed by flow cytometry. (A) CAFs were identified and excluded from further analysis through their expression of CD90 marker. CD90 negative cells, representing the cancer cells, were analysed. (B) DEAB was used as inhibitor for ALDH activity. This acted as negative control and allowed us to determine the gate position. (C) Cells with high-ALDH activity were populated in the gate.
