# Supplementary material for: A subtype of cancer-associated fibroblasts with lower expression of alpha-smooth muscle actin suppresses stemness through BMP4 in oral carcinoma
Source: Oncogenesis. 2018 Oct 5;7(10):78. doi: 10.1038/s41389-018-0087-x (PMC6172238; doi:10.1038/s41389-018-0087-x)
Supplement: Supplementary file 2 — Supplementary Methods [file 41389_2018_87_MOESM2_ESM.docx]

**Supplementary Materials and Methods**

**Generation of oral tumor organoids (OTOs) and establishment of cancer-associated fibroblasts cultures:**

Tumor tissues were washed with PBS containing antibiotic-antimycotic mix (Penicillin, Streptomycin, Gentamicin and Amphotericin B) (Cat. # 15240-062; Cat. # R01510; Thermo) at 2x concentration. Samples were transported to laboratory in serum free DMEM/F12K (1:1) media (cat # 11330-032; Thermo) (henceforth referred as DMEM/F12K) with antibiotic-antimycotic mix. Samples were minced into small pieces under sterile conditions and incubated for 2 hours in the presence of enzyme mix (Deoxyribonuclease 1 and Collagenase/Hyaluronidase Cat. # 17104-019; StemCell Technologies) in serum free DMEM-F12K at 37^0^C with intermittent mixing. After digestion, suspension was centrifuged at 500g for 10 seconds and resulting pellet with approximate size of 200μm tumor organoids were collected and plated over poly-D-lysine (Cat. # P6407; Sigma Aldrich), and collagen-I (Cat. # 4902; Stem cells Technologies) coated culture plates with DMEM/F12K containing Gentamicin, Amphotericin B and supplemented with 10% FBS (Cat. # 16140071; Thermo) and 1X Insulin Transferrin Selenium (ITS) (Cat. # 41400-045; Thermo). Fibroblasts grown from the tissues were dissociated and cultured separately in DMEM/F12K media with 10% FBS and 1X ITS; over freshly coated cell culture plates.

With this protocol, primary culture of GBC035 was established from primary tumor site of buccal mucosa. These cells were maintained in DMEM/F12K with 10% FBS and 1X ITS. Oral cancer cell line SCC-032 and SCC-029b was Kindly provided by Dr. Susanne M. Gollin, University of Pittusburgh, USA, which were established from the primary tumor sites of retromolar trigone and buccal mucosa respectively. These cell lines were maintained in MEM (Cat. # 11095-080; Thermo) with non-essential amino acid (Cat. # 11140050) and 10% FBS. For all co-culture experiments, CAFs and oral cancer cell lines were plated at a ratio of 3:1 for 5 days in DMEM/F12K media supplemented with 1% FBS and 0.1X ITS.

**Flow cytometry for cell surface markers and Aldefluor Assay:**

Single cell suspension was prepared using Accutase cell dissociation reagent (Cat. # A11105-01; Thermo) and re-suspended in FACS buffer (HBSS-buffer (Cat. # 14175-079; Thermo) supplemented with 0.5% FBS and 10mM HEPES (Cat. # 15630-080; Thermo)). As per the experiment, cells were incubated with CD90 BV421-conjugated (1:50, cat # 562556; BD), CD44 FITC-conjugated (1:10, Cat. # 555478; BD), CD24 BV421-conjugated (1:50, cat # 562789; BD) and EpCAM FITC-conjugated (1:10, cat # 347197; BD) in FACS buffer for 30 minutes at 4^0^C. After washing, cells were treated with Propidium Iodide (Sigma) to exclude dead cells. Isotype-matched IgG was tested for antibody specificity.

Aldefluor assay was performed using kit (Cat. # 01700, StemCell Technology) and according to manufacturer’s instructions. Briefly, 1x10^6^ cells/ml were suspended in Aldefluor buffer and incubated with 5μl/ml Aldefluor reagent BODIPY-aminoacetaldehyde (BAAA) for 60 minutes at 37^0^C. Diethyl aminobenzaldehyde (DEAB), an inhibitor of Aldefluor reagent, was used as negative control. Propidium iodide was used to exclude dead cells. CD90 BV421-conjugateed antibody was used to identify CAFs population in co-culture experiments. Flow cytometry data was acquired using BD Accuri C6 or BD FACS Aria Fusion cytometer and analysis was done with FCS Express 5 (DeNovo Software).

**Immunofluorescence staining:**

Cells were plated at the density of 10,000 cells per well as indicated, in 8-well chambered glass slides (Cat. # 0030742044, Eppendorf) freshly coated with poly-D-Lysine and Collagen-I. After 48 hours of plating, cells were fixed with 10% buffered formalin (Cat. #, H501128-4L; Sigma) for 30 minutes and washed with PBS contain glycine. Sorted cells were fixed and washed similarly and transferred over poly-D-Lysine coated slides and allowed to air dry. These immobilized cells were then re-hydrated by adding PBS. Rest of the protocol was same for both plated and sorted cells. Cells were then washed with ice-cold PBS containing glycine and permeabilized using 0.2% Tween-20 (Cat. # P1379; Sigma) at room temperature. Blocking was performed with 10% normal goat serum (Cat. # 5425S, Cell Signalling). Primary antibody against αSMA (cat no: ab7817 Abcam, 1:50), Vimentin (Cat. no: ab8978, Abcam, 1:200), BMP4 (Cat. # ab39973 Abcam, 1:200), Ki67 (Cat. # ab16667, Abcam, 1:100), CD90-conjugated with fluorescein isothiocyanate (FITC, cat # 562556, BD) and ALDH1A1 (Cat. # ab23375, Abcam) were used. Antibody was diluted in 0.2% BSA in PBS and incubated at 4^0^C, overnight. Next day, cells were washed and 1:500 dilution of secondary antibody conjugated with Alexa Fluor 488 (Cat. # 560173, BD) or Alexa Fluor 568 (A11019, Thermo) was used against mouse or rabbit IgG, for 2 hrs at room temperature. Isotype-matched IgG was used to test antibody specificity. Cells were washed with PBS and mounted using DAPI containing mounting medium (ProLong® Diamond Antifade Mountant (Cat. # P36962 Thermo). Stained cells were examined and images were acquired using EVOS-FL (Thermo) microscope.

Semi-quantitative score for αSMA was determined by taking into consideration both cellularity and intensity of αSMA-positive ultrastructure formation or stress-fibers (semi-quantitative score = cellularity × intensity). Cellularity (based on the percentage of positive cells in 3 independent fields) was scored as follows: a score of 1 for less than 10% cellularity, a score of 2 for 10%–30% cellularity, a score of 3 for 31-50% cellularity and a score of 4 for more than 50% cellularity. Intensity was scored as follows: a score of 3 equals to strong intensity, a score of 2 equals to moderate intensity, and a score of 1 equals to weak intensity. ALDH1A1 and Vimentin immunofluorescence staining for sorted cells was quantified using ImageJ software (Nature methods 9(7): 676-682). From each image, integrated density of every cell was calculated after subtracting the background fluorescence. Total 10 images were analyzed for the mean integrated density of eGFP positive and negative cells.

**RNA preparation, gene expression microarray, real-time PCR and bioinformatics:**

CAFs were plated on poly-D-Lysine and Collagen-I coated 100 mm tissue culture dishes. Fresh media was added 24 hour before RNA isolation. RNA was isolated by RNeasy mini kit (Cat. # 74104, Qiagen) following manufacturer’s instructions.

For microarray analysis, quality of RNA was assessed on Agilent Bioanalyser 2100 (Agilent Technologies) by using Agilent RNA nano kit (Cat # 5067-1511, Agilent Technologies) according to the manufacturer’s instructions. 500ng of RNA was amplified using Illumina ‘Total Prep-RNA Amplification Kit’ (Cat. # AMIL1791, Thermo) as per manufacturer’s instruction. Total 750ng of cRNA was labelled with Cy3-Streptavidin (Cat. # PA43001; GE Healthcare) and hybridized to Illumina Human HT-12 v4.0 Expression BeadChip (Illumina) with manufacturer’s protocol. Chip was scanned with illumina iScan systems (Illumina). Unsupervised clustering was performed using 1000 most variably expressed probes from gene expression data for clustering the samples. Differential gene expression analysis was done using Bioconductor package limma and R statistical software (version 3.2.3; Wooden Christmas-Tree) (**(#limma - 10.1093/nar/gkv007)**. DAVID functional annotation tool (version 6.8) was used to identify enriched GO terms and pathways (**#DAVID - 10.1038/nprot.2008.211)**. Protein-protein interaction networks were identified using STRING database (version 10) **(#STRING - 10.1093/nar/gku1003).**

For real-time qPCR analysis, 450ng of RNA was transcribed into cDNA using Verso cDNA synthesis kit (Cat. # AB1453A, Thermo) following manufacturer's instructions. Real-Time PCR was performed on ABI PRISM 7900HT Fast Real Time PCR System (Applied Biosystems) using KAPA SYBR FAST qPCR Kit (Cat. # KK4601, Kapa Biosystems). The expression levels of genes were normalized with β−actin and defined as ΔΔCt = [ΔCt*_sample_* - ΔCt*_actin_*]. Fold change of the genes were calculated by 2^−ΔΔCT^ method **(Methods. 2001 Dec;25(4):402-8)**.

| **List of primers used in the study** | | |
| --- | --- | --- |
| **Sl. #** | **Gene** | **Primer sequence (5’-3’)** |
| 1 | aSMA_F | TGGGTGACGAAGCACAGAGC |
|  | aSMA_R | CTTCAGGGGCAACACGAAGC |
| 2 | FSP1_F | AGTACGTGTTGATCCATGACTG |
|  | FSP1_R | AACTTGTCACCCTCTTTGCC |
| 3 | PDGFRb_F | GGACACCATGCGGCTTCC |
|  | PDGFRb_R | CCCTGAGAGATCTGTGGTTCC |
| 4 | CCL2_F | GACCACCTGGACAAGCAAAC |
|  | CCL2_R | TGTTCTGGGGAAAGCTAGGGG |
| 5 | TGFb1_F | GGTGTCTCAGTATCCCAGGG |
|  | TGFb1_R | AGACGATCTCTCTCCGACC |
| 6 | Oct4_F | GACAGGGGGAGGGGAGGAGCTAGG |
|  | Oct4_R | CTTCCCTCCAACCAGTTGCCCCAAAC |
| 7 | Sox2_F | AGTATCAGGAGTTGTCAAGGC |
|  | Sox2_R | AGTCCTAGTCTTAAAGAGGCA |
| 8 | Nanog_F | CAGCCCCGATTCTTCCACCAGTCCC |
|  | Nanog_R | CGGAAGATTCCCAGTCGGGTTCACC |
| 9 | ALDH1A1_F | GATGCCGACTTGGACAATGC |
|  | ALDH1A1_R | TCTTAGCCCGCTCAACACTC |
| 10 | ITGB1_F | CGCGGAACAGCAGGCCCGAG |
|  | ITGB1_R | TGAGCAAACACACAGCAAAC |
| 11 | ITGA3_F | GCTACATGATTCAGCGCAAGG |
|  | ITGA3_R | GGATGAAGCTGCCTACCTGC |
| 12 | EYA1_F | CCCTTCCAACAGACCATACC |
|  | EYA1_R | AGCTTGTTGCATTCCTGTGG |
| 13 | MYC _F | GGACCCGCTTCTCTGAAAGG |
|  | MYC_R | TAACGTTGAGGGGCATCGTC |
| 14 | VEGFA_F | GTCCTGGAGCGTGTACGTTG |
|  | VEGFA_R | ACAAATGCTTTCTCCGCTCTG |
| 15 | FOXF1_F | CCAGCGAGTTCATGTTCGAGG |
|  | FOXF1_R | GTTGAAGCCGAGCCCGTTC |
| 16 | RUNX2_F | GCAAGCAGTATTTACAACAGAGG |
|  | RUNX2_R | ACTGTGCTGAAGAGGCTGTT |

**Collagen contraction assay**

Collagen contraction assay was performed as described previously (Nature Cell Biology, 2013 (15): 637–646). In short, 1x10^5^ CAFs were mixed with Collagen-I (Cat. # A10483-01, Thermo) and Matrigel (Cat. # 354234, Corning) mix at the concentration of 4.6mg/ml and 2.2mg/ml respectively. CAFs and matrix mix was plated in the wells of 24 well plate. Once the gel was set, DMEM/F12K media supplemented with 10% FBS and 1x ITS was added on top. Gel was released from the sides of plate with the help of a fine tip, to allow contraction. Image of the plate was acquired in Chemidoc imaging system (BioRad). Area of the gels was quantified using ImageJ software using oval tool and extrapolated to the surface area of a well of 24 well plate (200 mm^2^).

**Sphere Formation Assay**

5 cells/μl of SCC-032 were resupended in MEM media containing 20ng/ml EGF (Cat. # PHG0311; Thermo), bFGF (Cat. # PHG0261; Thermo), 1x B-27 (Cat. # 12587010; Thermo) and 0.4 μg/ml hydrocortisone (Cat. # H6909; Merck) along with 1.25% geltrex (Cat. # A14132-01, Thermo). BMP4 (Cat. # PHC9534; Thermo) was added at concentrations of 20 and 50 ng/ml, mixed well and plated in multiple wells of 24 well ultra-low attachment plate (Corning). Growth factors were supplemented every third day along with fresh BMP4. Sphere formation was continued for 7 days. Spheres with diameter of 60 µm or more were considered for counting the number of spheres formed and calculating their average size.

**Senescence associated β-gal assay:**

To test the presence of senescent cells in growing cultures of CAFs, cells were cultured in 8-well chamber slide (10,000 cells per well) in regular culture medium. After 48 hr of culture, presence of senescent cells was evaluated by cellular senescence assay kit (Catalog number CBA-230; Cell Biolabs, Inc) following manufacturer’s instructions. Images were acquired using 100x magnification in bright-field microscope (Olympus).

**Immunohistochemical Staining of Ki67, ALDH1A1 and αSMA for gingivobuccal-oral cancer tissues:**

Treatment naïve, human gingivobuccal oral tumor tissues were immunostained for ALDH1A1, Ki67 and αSMA. Tissue slides were deparaffinized by heating at 62°C for 1 hour and immersing thrice in 100% xylene, followed by rehydrating with decreasing concentrations (100%, 90%, 80%, 70%) of ethyl alcohol. Slides were rinsed in cold water and subjected to antigen retrieval for 10 minutes by boiling in pressure cooker in 10mM sodium citrate buffer (pH 6.0) with 0.05% Tween 20. Sections were cooled to room temperature. After rinsing with PBS, the endogenous peroxidase activity was quenched by dipping tissue in 3% H_2_O_2_ in PBS for 10 minutes. Slides were rinsed with PBS and blocked with blocking serum (Cat. # 7200, Vector Laboratories) followed by overnight incubation with primary antibody (αSMA, 1:50; ALDH1A1 1:500 or Ki67, 1:100) at 4^0^C. Slides were rinsed with PBS and incubated with secondary antibody (Vector Laboratories) for 30 minutes at room temperature followed by incubation with avidin–biotin–horseradish peroxidase complex reagent for 30 minutes at room temperature (Vector Laboratories). Peroxidase substrate kit with 3,3′-diaminobenzidine (DAB) (Cat. # SK-4100, Vector Laboratories) as chromogen was added over the tissue sections till the color developed. Reactions for all the samples were stopped after same duration of incubation. Immediately, slides were rinsed with distilled water and lightly counterstained with hematoxylin (Cat. # 61826701251730; Merck). Sections were dehydrated by immersing in increasing concentrations of ethyl alcohol; finally to 100%. Slides were mounted in DPX mounting media (Cat. # H-5000, Vector Laboratories). Images were acquired using bright-field microscopes (Olympus) with MSHOT microscope camera and image analysis software. Pathologist (IA) examined all the slides in blinded manner and scored based on the staining of αSMA in tumor stroma. The semiquantitative score for αSMA staining was given by keeping both cellularity and intensity of expression into consideration (semi-quantitative score = cellularity × intensity). Cellularity was scored as follows: a score of 3 was given for equals to or greater than 66% cellularity, a score of 2 was given for 34%–65% cellularity, and a score of 1 was given to less than 33% cellularity. Intensity was scored as follows: a score of 3 was for strong intensity, a score of 2 was for moderate intensity and a score of 1 was given for weak intensity. Ki67 and ALDH1A1 staining was scored by focusing in the positively stained areas of tumor lobes using 200x magnification. We counted all positive cells and calculated the %positivity by comparing its fraction from total number of cells in the same observed field. Minimum of 300 cells were counted for each sample with ImageJ software.

**siRNA mediated knockdown:**

Cells were transfected with 50nM of siRNA of BMP4 or non-targeting siRNA negative control (Eurogentec) for 48 hour in low serum media using INTERFERin kit (Cat# 409-10; Polypus) following manufacturer’s instructions.
