# Supplementary material for: A subtype of cancer-associated fibroblasts with lower expression of alpha-smooth muscle actin suppresses stemness through BMP4 in oral carcinoma
Source: Oncogenesis. 2018 Oct 5;7(10):78. doi: 10.1038/s41389-018-0087-x (PMC6172238; doi:10.1038/s41389-018-0087-x)
Supplement: Supplementary file 3 — Supplementary Table 1 [file 41389_2018_87_MOESM3_ESM.docx]

**Supplementary Table 1:** List of differentially expressed genes in C1-type CAFs verses C2-Type CAFs (Probes with fold change-more than 2 and adjusted *P* value less than 0.05 were shortlisted)

| **Probe** | **SYMBOL** | **FC** |
| --- | --- | --- |
| ILMN_1785071 | SEPP1 | 6.262301214 |
| ILMN_1694778 | LOC646723 | 0.029650909 |
| ILMN_1789639 | FMOD | 8.217991584 |
| ILMN_2041222 | FLJ40504 | 0.100675382 |
| ILMN_1718565 | CDKN1C | 2.697546182 |
| ILMN_1739640 | DCHS1 | 2.33613645 |
| ILMN_1803094 | PDGFD | 6.330961662 |
| ILMN_1730504 | AGPAT4 | 2.304340493 |
| ILMN_3247023 | FLJ22536 | 4.310284344 |
| ILMN_2376859 | PDGFD | 7.092837816 |
| ILMN_1767362 | ADAMTS6 | 0.279417766 |
| ILMN_1755215 | SAMD5 | 7.547578811 |
| ILMN_1738989 | GOLSYN | 5.262859298 |
| ILMN_1722524 | GALNT14 | 2.229402306 |
| ILMN_1770228 | KRT34 | 0.04690977 |
| ILMN_1784630 | KBTBD11 | 10.78661449 |
| ILMN_1799026 | PCDH18 | 5.280253074 |
| ILMN_1699867 | IGF2 | 7.310236072 |
| ILMN_1718866 | C5orf46 | 0.056189152 |
| ILMN_2229877 | PCDH18 | 4.500383154 |
| ILMN_1658917 | SLC1A1 | 0.238818941 |
| ILMN_1701025 | EPHX1 | 3.651980959 |
| ILMN_2066151 | TEK | 0.092328313 |
| ILMN_1742824 | SPATA13 | 4.692390668 |
| ILMN_1801205 | GPNMB | 2.996238661 |
| ILMN_2413956 | IGF2 | 9.373918273 |
| ILMN_1742260 | SSFA2 | 2.103123919 |
| ILMN_1751576 | TEK | 0.1013 |
| ILMN_1667050 | PRPS1 | 0.298972457 |
| ILMN_1756312 | SEMA7A | 0.263604862 |
| ILMN_1764082 | MBOAT1 | 4.694929395 |
| ILMN_1740900 | BMP4 | 7.910278926 |
| ILMN_1719543 | MAF | 5.24325702 |
| ILMN_1723874 | MRPS6 | 2.267006486 |
| ILMN_1709683 | RASSF2 | 5.901554068 |
| ILMN_1687533 | SEMA4D | 3.352858254 |
| ILMN_2225548 | ZNF521 | 3.225438712 |
| ILMN_1669046 | FOXQ1 | 4.488544251 |
| ILMN_1815745 | SOX4 | 4.440713034 |
| ILMN_1659158 | SAMD3 | 0.493385487 |
| ILMN_1807969 | SNCAIP | 4.152488057 |
| ILMN_1794825 | ALDH3A2 | 3.553712555 |
| ILMN_1797735 | C1orf21 | 3.537101916 |
| ILMN_2407389 | GPNMB | 2.92108119 |
| ILMN_2304186 | MITF | 2.09778504 |
| ILMN_3238676 | ULBP2 | 0.300120806 |
| ILMN_1742332 | KCTD12 | 3.431248746 |
| ILMN_2169152 | SRGN | 0.059232948 |
| ILMN_1732923 | SIPA1L2 | 10.3009834 |
| ILMN_1668312 | SLC2A9 | 2.46318225 |
| ILMN_1772612 | ANGPTL2 | 5.709565127 |
| ILMN_1680973 | FOXF1 | 4.173462845 |
| ILMN_2371458 | CXCR7 | 12.53567447 |
| ILMN_1689431 | APCDD1L | 0.217925338 |
| ILMN_2352303 | RASSF2 | 4.756102379 |
| ILMN_2044645 | CGB1 | 0.461938194 |
| ILMN_1798926 | SOCS2 | 5.095811264 |
| ILMN_1725338 | CLDN23 | 7.990355641 |
| ILMN_1812795 | RUNX1T1 | 2.294928184 |
| ILMN_1754076 | CACNA2D3 | 5.558095117 |
| ILMN_1696048 | C13orf33 | 0.090609993 |
| ILMN_1760347 | SRGN | 0.065781942 |
| ILMN_1659761 | SNX29 | 2.279596011 |
| ILMN_1758938 | SLC31A2 | 0.452876581 |
| ILMN_1813685 | RAB7L1 | 2.074969565 |
| ILMN_2401641 | ALDH3A2 | 3.170850469 |
| ILMN_1807554 | EYA1 | 6.535726642 |
| ILMN_1715669 | TP53I11 | 2.296459255 |
| ILMN_2399769 | GPR177 | 3.047415459 |
| ILMN_1715662 | CCDC80 | 0.383616849 |
| ILMN_2131861 | SOCS2 | 5.124356054 |
| ILMN_1809813 | PGF | 6.137579993 |
| ILMN_3209399 | LOC399965 | 0.429218444 |
| ILMN_1704154 | TNFRSF19 | 9.395307707 |
| ILMN_2359601 | CAMK2G | 2.144136556 |
| ILMN_2174127 | DCBLD2 | 0.481875247 |
| ILMN_1787265 | ZNF503 | 2.399730478 |
| ILMN_1711628 | LOC728951 | 0.271413394 |
| ILMN_1683664 | LOC650369 | 0.462132447 |
| ILMN_1658926 | NOTCH3 | 0.133149012 |
| ILMN_1656826 | SH3RF1 | 0.454036096 |
| ILMN_2075757 | LRIG3 | 2.239124753 |
| ILMN_1784454 | ITGB1 | 0.373867186 |
| ILMN_1709795 | RAC2 | 0.095435184 |
| ILMN_1808999 | ARHGEF19 | 2.166630611 |
| ILMN_3248701 | LOC644350 | 0.190981482 |
| ILMN_1735499 | DCBLD2 | 0.482637776 |
| ILMN_1766914 | MFAP4 | 5.073240199 |
| ILMN_1785290 | DOK6 | 2.822160188 |
| ILMN_1715748 | FLNC | 0.429723504 |
| ILMN_3248758 | LOC728934 | 0.313340478 |
| ILMN_1794501 | HAS3 | 0.451205589 |
| ILMN_2114720 | SLPI | 4.039491385 |
| ILMN_1781626 | C1S | 3.22490557 |
| ILMN_1656111 | MYLIP | 3.823626194 |
| ILMN_1798360 | CXCR7 | 13.3932633 |
| ILMN_1665219 | LTBP4 | 4.836691351 |
| ILMN_1819384 | NNN | 0.390737803 |
| ILMN_2379560 | CDC14B | 2.330945163 |
| ILMN_1744381 | SERPINE1 | 0.219013621 |
| ILMN_1761247 | PIR | 3.10725739 |
| ILMN_2129161 | LRRC32 | 0.107121081 |
| ILMN_1679754 | ADRA1B | 3.066334034 |
| ILMN_2096784 | TFAP2C | 5.836267046 |
| ILMN_2224444 | SNX25 | 0.456675423 |
| ILMN_2110908 | MYC | 0.488613605 |
| ILMN_1792409 | AMOT | 3.123771025 |
| ILMN_1743275 | SH3RF3 | 2.132686343 |
| ILMN_2179778 | PHLDB2 | 0.444392803 |
| ILMN_1766411 | AP1S2 | 2.454945302 |
| ILMN_1667564 | ALDH3A2 | 2.231984376 |
| ILMN_1803956 | BOC | 2.018975602 |
| ILMN_2336609 | SYTL2 | 4.169583797 |
| ILMN_3251708 | LOC728946 | 0.203285612 |
| ILMN_1714167 | CYB5A | 2.309066912 |
| ILMN_1791280 | HSPB8 | 0.302107697 |
| ILMN_2062701 | GAS1 | 4.658481957 |
| ILMN_1685397 | ITGA3 | 0.396801845 |
| ILMN_1707652 | KRTAP1-5 | 0.205305992 |
| ILMN_1782922 | PDE4B | 2.986181478 |
| ILMN_2189306 | ROBO2 | 6.300448884 |
| ILMN_3194087 | HSPC157 | 2.00596245 |
| ILMN_1869897 | NNN | 0.385421047 |
| ILMN_1720513 | SETBP1 | 3.666180583 |
| ILMN_2373062 | RHBDF2 | 0.317068907 |
| ILMN_1666022 | TNFRSF10D | 0.30554843 |
| ILMN_1660067 | LOC728285 | 0.17241135 |
| ILMN_1731374 | CPE | 7.669431234 |
| ILMN_1765109 | TNFRSF25 | 0.404002614 |
| ILMN_1658494 | C13orf15 | 14.96056868 |
| ILMN_3305849 | LOC728431 | 2.139379717 |
| ILMN_1681558 | LOC728956 | 0.31456026 |
| ILMN_1766499 | HSPA2 | 2.669870285 |
| ILMN_1758548 | NEK7 | 0.400955787 |
| ILMN_1716651 | RUNX2 | 2.546895517 |
| ILMN_1803882 | VEGFA | 0.344561958 |
| ILMN_1744403 | KCNIP3 | 3.695938639 |
| ILMN_1719792 | PHLDB2 | 0.382429977 |
| ILMN_1736178 | AEBP1 | 2.725204166 |
| ILMN_1763852 | ACACB | 2.313079388 |
| ILMN_1699071 | C21orf7 | 0.336784977 |
| ILMN_1682332 | GYPC | 2.153837405 |
| ILMN_1653836 | C11orf41 | 0.300423697 |
| ILMN_3294642 | LOC392787 | 0.40649758 |
| ILMN_1668039 | GYPC | 2.236718062 |
| ILMN_1781374 | TUFT1 | 0.403093897 |
| ILMN_1683891 | TFAP2C | 5.652760783 |
| ILMN_1682929 | SYTL2 | 4.391708663 |
| ILMN_2209260 | KRTAP1-3 | 0.216147931 |
| ILMN_1803338 | CCDC80 | 0.418136824 |
| ILMN_2340259 | PDE4B | 5.292638324 |
| ILMN_1758315 | SLC9A9 | 2.829933362 |
| ILMN_1741954 | SMYD3 | 0.326318567 |
| ILMN_1669119 | LOC728946 | 0.205392374 |
| ILMN_1727135 | FIBCD1 | 0.44240205 |
| ILMN_1780132 | PELI2 | 3.735113786 |
