# Supplementary material for: A subtype of cancer-associated fibroblasts with lower expression of alpha-smooth muscle actin suppresses stemness through BMP4 in oral carcinoma
Source: Oncogenesis. 2018 Oct 5;7(10):78. doi: 10.1038/s41389-018-0087-x (PMC6172238; doi:10.1038/s41389-018-0087-x)
Supplement: Supplementary file 4 — Supplementary Table 2 [file 41389_2018_87_MOESM4_ESM.docx]

| **Supplementary Table 2:** Clinical characteristics of patients | | | | | | | |
| --- | --- | --- | --- | --- | --- | --- | --- |
| **Tumor Sl.No.** | **TNM Status** | **Grade** | **Age** | **Gender** | **Tobacco use/Smoking** | **aSMA Score** | **ALDH Score** |
| 1 | pT4aN0M0 | MD | 56 | Male | No | 1 | High |
| 2 | pT4bN1M0 | PD | 43 | Male | No | 1 | Low |
| 3 | pT4bN2M0 | PD | 61 | Female | Yes | 1 | Low |
| 4 | pT2N0M0 | WD | 68 | Male | Yes | 1 | High |
| 5 | pT4aN2bM0 | PD | 63 | Female | No | 1 | NA |
| 6 | pT2N0M0 | MD | 80 | Female | NA | 2 | Low |
| 7 | pT4aN0M0 | PD | 49 | Male | Yes | 2 | NA |
| 8 | pT4aN0M0 | WD | 49 | Male | Yes | 2 | Low |
| 9 | pT4aN2bM0 | MD | 53 | Male | Yes | 2 | High |
| 10 | pT2N0M0 | MD | 62 | Female | NA | 0 | Low |
| 11 | pT4N2bM0 | MD | 48 | Male | NA | 0 | Low |
| 12 | pT2N0M0 | MD | 70 | Male | NA | 1 | Low |
| 13 | pT2N0M0 | WD | 50 | Male | NA | 1 | Low |
| 14 | pT2N0M0 | MD | 32 | Male | NA | 2 | Low |
| 15 | pT2N2bM0 | PD | 53 | Female | NA | 3 | High |
| 16 | pT2N1M0 | WD | 65 | Male | No | 2 | Low |
| 17 | pT2N0M0 | MD | 59 | Male | NA | 2 | NA |
| 18 | pT4aN0M0 | WD | 55 | Male | NA | 4 | Low |
| 19 | pT4aN0M0 | WD | 60 | Female | Yes | 4 | Low |
| 20 | pT4aN2bM0 | PD | 68 | Male | No | 4 | Low |
| 21 | pT2N2bM0 | MD | 36 | Male | NA | 4 | High |
| 22 | pT4aN1M0 | WD | 52 | Male | Yes | 4 | Low |
| 23 | pT1N1M0 | MD | 44 | Male | Yes | 6 | High |
| 24 | pT2N2bM0 | PD | 50 | Male | Yes | 6 | Low |
| 25 | pT4aN0M0 | WD | 69 | Male | No | 6 | Low |
| 26 | pT2N2M0 | WD | 46 | Male | No | 6 | Low |
| 27 | pT4aN0M0 | MD | 39 | Male | Yes | 6 | High |
| 28 | pT2N2bM0 | PD | 36 | Male | No | 6 | High |
| 29 | pT1N0M0 | MD | 63 | Male | Yes | 6 | High |
| 30 | pT4bN0M0 | MD | 47 | Female | No | 6 | Low |
| 31 | pT2N0M0 | MD | 44 | Male | No | 8 | High |
| 32 | pT4aN1M0 | PD | 71 | Male | No | 9 | High |
| 33 | pT2N0M0 | MD | 45 | Female | No | 9 | High |
| 34 | pT4aN1M0 | MD | 50 | Male | No | 9 | High |
| 35 | pT4bN0M0 | MD | 42 | Male | Yes | 9 | High |
| 36 | pT3N2bM0 | WD | 41 | Male | Yes | 9 | Low |
| 37 | pT4aN0M0 | NA | 59 | Male | Yes | 9 | High |
| 38 | pT2N1M0 | WD | 66 | Female | NA | 12 | Low |
| 39 | pT3N2cM0 | MD | 46 | Male | Yes | 12 | High |
| 40 | pT4bN2bM0 | MD | 35 | Male | Yes | 12 | High |
| 41 | pT3N2bM0 | WD | 42 | Male | No | 12 | High |
| 42 | pT4aN1M0 | MD | 55 | Female | No | 12 | Low |
| 43 | pT4aN2bM0 | MD | 40 | Female | NA | 8 | High |
| 44 | pT4aN0M0 | MD | 60 | Female | NA | 8 | High |
| 45 | pT4aN2bM0 | WD | 50 | Male | NA | 12 | High |
| 46 | pT4aN2CM0 | MD | 32 | Male | NA | 12 | NA |
