# Supplementary figures and images for: A subtype of cancer-associated fibroblasts with lower expression of alpha-smooth muscle actin suppresses stemness through BMP4 in oral carcinoma
Source: Oncogenesis. 2018 Oct 5;7(10):78. doi: 10.1038/s41389-018-0087-x (PMC6172238; doi:10.1038/s41389-018-0087-x)

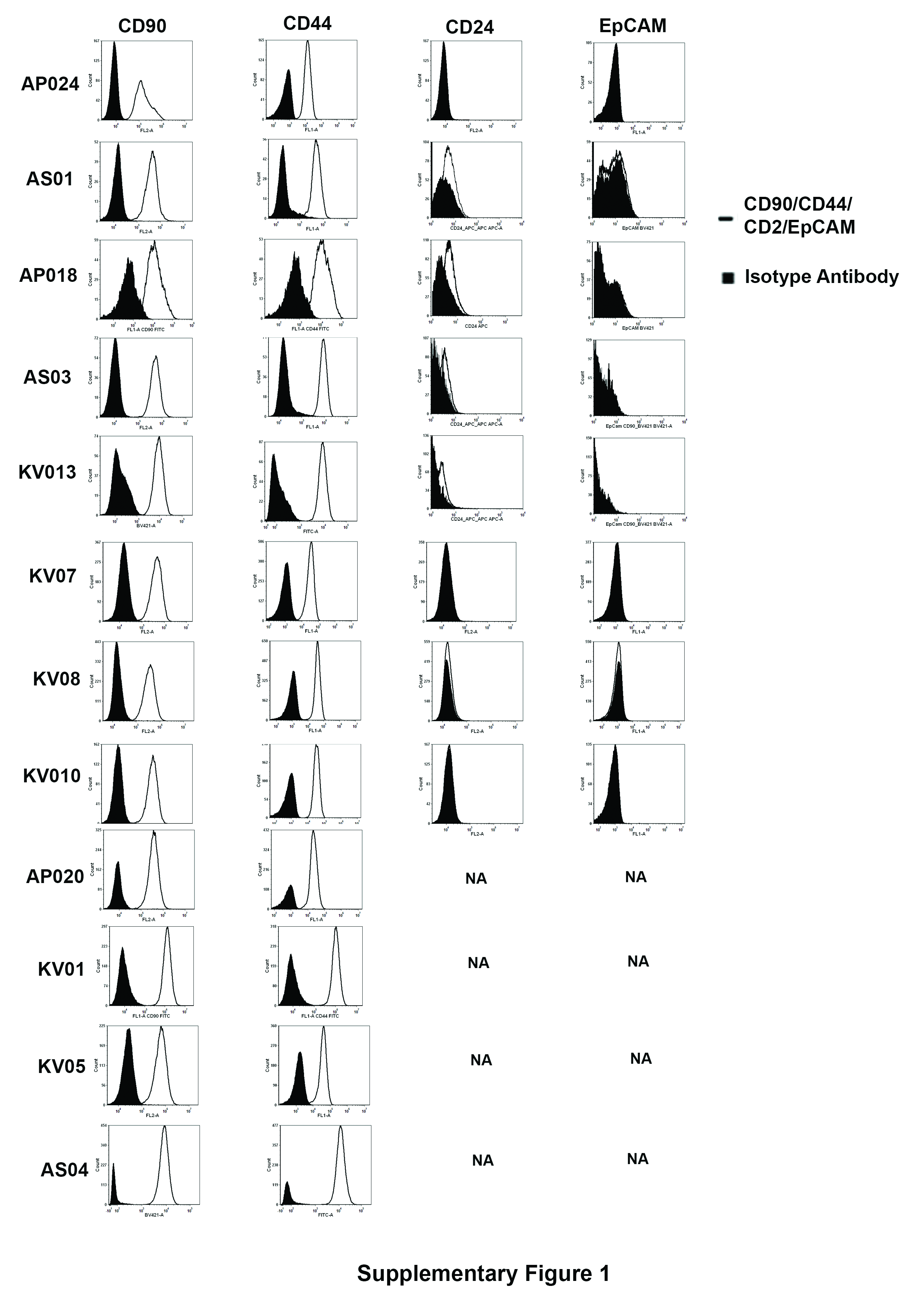

Supplement: Supplementary file 5 — Supplementary Figure S1 [file 41389_2018_87_MOESM5_ESM.tif]

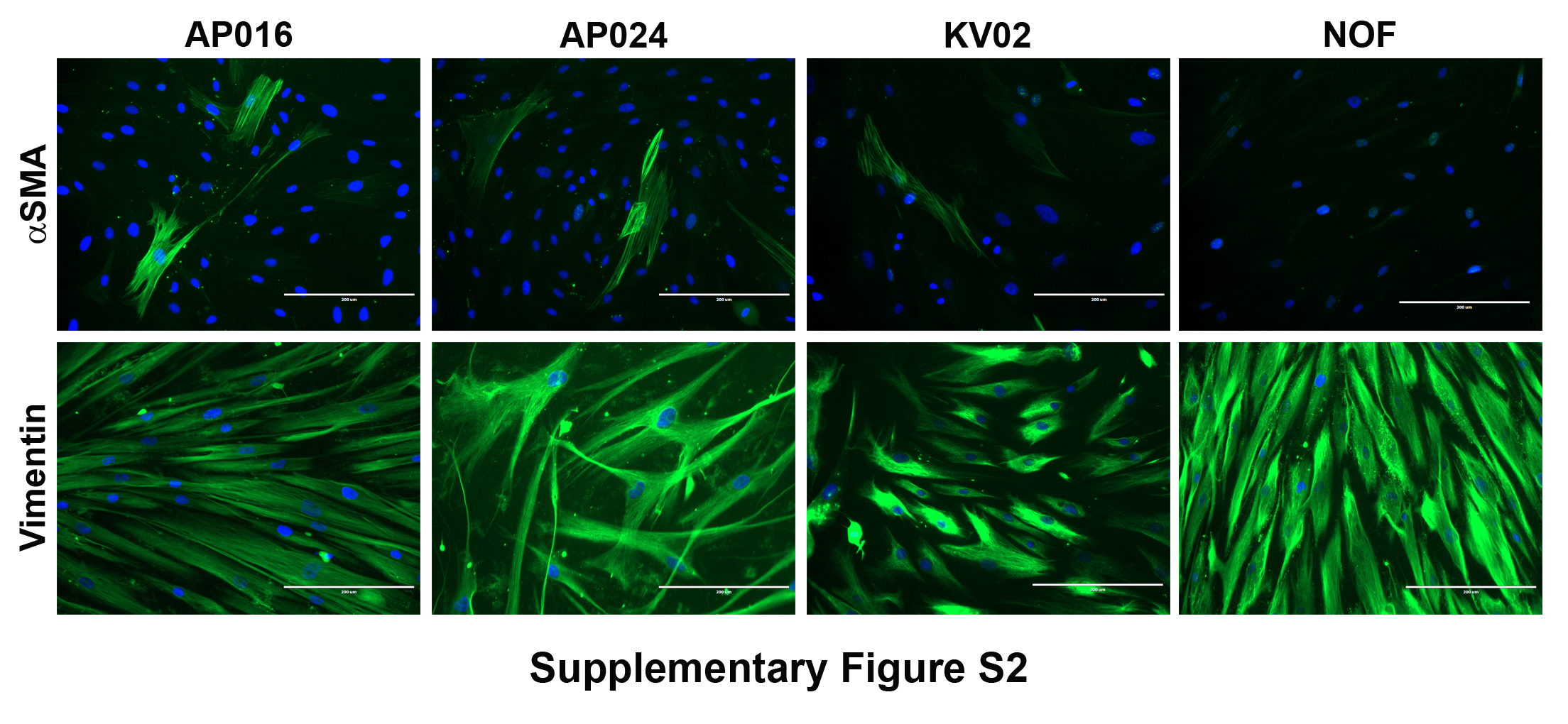

Supplement: Supplementary file 6 — Supplementary Figure S2 [file 41389_2018_87_MOESM6_ESM.tif]

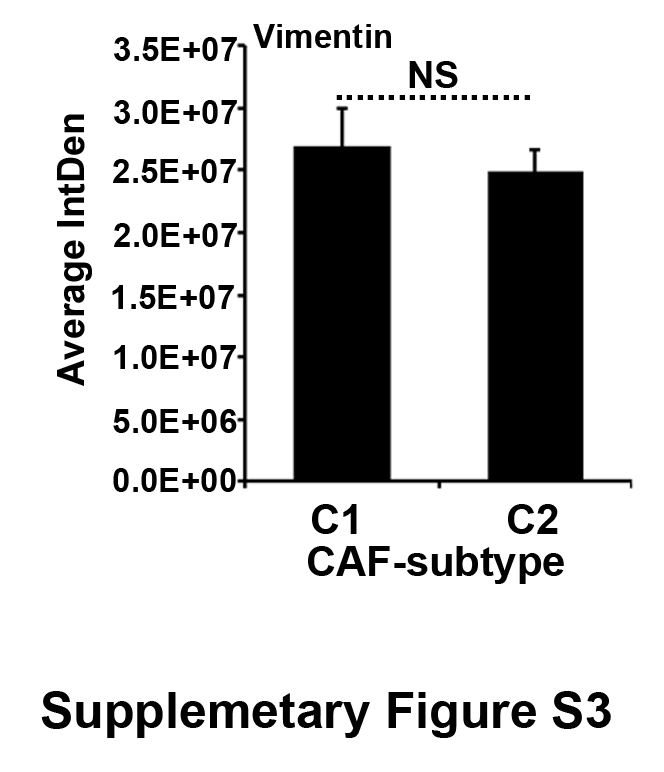

Supplement: Supplementary file 7 — Supplementary Figure S3 [file 41389_2018_87_MOESM7_ESM.tif]

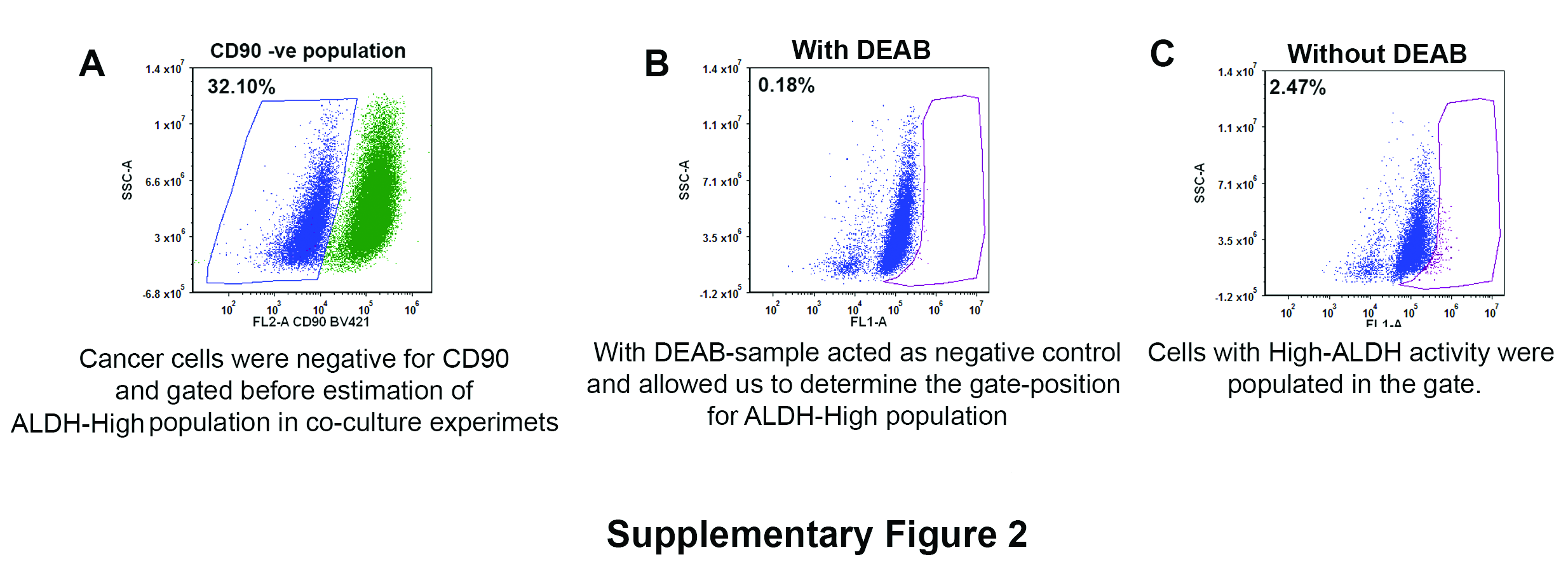

Supplement: Supplementary file 8 — Supplementary Figure S4 [file 41389_2018_87_MOESM8_ESM.tif]
